# *In Silico* Analysis of the Fucosylation-Associated Genome of the Human Blood Fluke *Schistosoma mansoni*: Cloning and Characterization of the Fucosyltransferase Multigene Family

**DOI:** 10.1371/journal.pone.0063299

**Published:** 2013-05-16

**Authors:** Nathan A. Peterson, Tavis K. Anderson, Timothy P. Yoshino

**Affiliations:** 1 Department of Pathobiological Sciences, University of Wisconsin, Madison, Wisconsin, United States of America; 2 Department of Entomology, University of Wisconsin, Madison, Wisconsin, United States of America; 3 Virus and Prion Research Unit, National Animal Disease Center, United States Department of Agriculture, Agricultural Research Service, Ames, Iowa, United States of America; Queensland Institute of Medical Research, Australia

## Abstract

Fucosylated glycans of the parasitic flatworm *Schistosoma mansoni* play key roles in its development and immunobiology. In the present study we used a genome-wide homology-based bioinformatics approach to search for genes that contribute to fucosylated glycan expression in *S. mansoni*, specifically the α2-, α3-, and α6-fucosyltransferases (FucTs), which transfer L-fucose from a GDP-L-fucose donor to an oligosaccharide acceptor. We identified and *in silico* characterized several novel schistosome FucT homologs, including six α3-FucTs and six α6-FucTs, as well as two protein O-FucTs that catalyze the unrelated transfer of L-fucose to serine and threonine residues of epidermal growth factor- and thrombospondin-type repeats. No α2-FucTs were observed. Primary sequence analyses identified key conserved FucT motifs as well as characteristic transmembrane domains, consistent with their putative roles as fucosyltransferases. Most genes exhibit alternative splicing, with multiple transcript variants generated. A phylogenetic analysis demonstrated that schistosome α3- and α6-FucTs form monophyletic clades within their respective gene families, suggesting multiple gene duplications following the separation of the schistosome lineage from the main evolutionary tree. Quantitative decreases in steady-state transcript levels of some FucTs during early larval development suggest a possible mechanism for differential expression of fucosylated glycans in schistosomes. This study systematically identifies the complete repertoire of FucT homologs in *S. mansoni* and provides fundamental information regarding their genomic organization, genetic variation, developmental expression, and evolutionary history.

## Introduction

Chronic schistosomiasis in mammalian hosts, including humans, results from granulomatous inflammation in response to parasite eggs that accumulate in host tissues [1]. Previous studies in *Schistosoma mansoni* suggest that surface-expressed and secreted/excreted carbohydrates are key elements that drive this pathogenesis, with oligosaccharides playing roles in egg sequestration, Th2 immune biasing, granuloma formation and modulation, and strong antibody responses in human hosts [2], [3]. Likewise, in snail intermediate hosts, carbohydrates serve as ligands to plasma-associated lectins and mediate hemocyte encapsulation and cytotoxic reactive oxygen species responses [4], [5]. A common determinant in many of these host-parasite interactions is the deoxyhexose sugar L-fucose, which comprises as much as 40% of the total structural carbohydrates in larvae of *S. mansoni* [6].

Alpha2- and α3-linked fucoses are major constituents of a diverse group of immunologically important LacdiNAc (LDN; GalNAcβ1-4GlcNAc)-derived glycotopes, including F-LDN, LDN-F, F-LDN-F, LDN-DF and DF-LDN-DF, as well as the Lewis X glycotope, which constitute the non-reducing ends of protein- and lipid-conjugated oligosaccharides in both mammalian and snail host-associated developmental stages [7]–[10]. Many aspects of schistosome pathogenesis mentioned above are attributable to these glycotopes. Additionally, α3- and α6-linked fucoses occur alone or in combination as substituents of the chitobiose core (-GlcNAcβ1-4GlcNAcβ1-) in paucimannosidic and complex type N-glycans [11]. Such core modifications, especially α3-fucosylation, induce strong allergic responses in mammalian hosts and account for the interspecific immunological cross-reactivity observed among plant, insect, and helminth glycoproteins [12], [13]. Importantly, studies indicate that the expression of fucosylated glycotopes in *S. mansoni* is stage- and gender-specifically regulated [7], [9], [10], [14], yet the mechanisms of this regulation, including the underlying enzymatic machinery, remains largely unknown. To better understand the developmental expression of immunologically important fucosylated glycans in *S. mansoni*, basic information is needed regarding the repertoire of Golgi-localized fucosyltransferases (FucTs), specifically the α2-, α3- and α6-FucTs, which transfer L-fucose from a guanosine diphosphate (GDP)-L-fucose donor to an oligosaccharide acceptor, creating α2, α3, or α6 linkages.

While α2-, α3-, and α6-linked fucoses are prevalent in schistosome fucoconjugates and all three fucosylation activities have been observed in extracts of various developmental stages [15]–[18], only α3-FucTs have been described in *S. mansoni*. These include homologs “*SmFuct*” [19] and “*SmFucTA*” [20], which are herein referred to as *FucT-VII* and *FucTA*, respectively. More recently, Fitzpatrick et al. [21] identified eight distinct Pfam-annotated putative α3-FucT genes, including one corresponding to *FucTA*, in the *S. mansoni* GeneDB database. However, to date, the full extent of the α3-FucT multigene family in *S. mansoni* is still unknown, and most of these predicted genes have yet to be substantively characterized. Furthermore, nothing is known about the α2- and α6-FucT genes in *S. mansoni* despite the wide distribution and abundance of glycans displaying α2- and α6-linked fucose. In the present study, we used a homology-based genome-wide bioinformatics approach to identify and *in silico* characterize the complete repertoire of schistosome FucT homologs. In addition to the α2-, α3- and α6- FucTs, our investigation included the protein O-FucTs, which are not associated with glycotope expression but instead transfer L-fucose to serine and threonine residues of epidermal growth factor- and thrombospondin-type repeats. To our knowledge, this is the most comprehensive study to date regarding the genomic organization, alternative splicing, and molecular phylogenetics of the FucTs in *S. mansoni*. Additionally, given the prominence of fucosylated glycans expressed at the host-parasite interface and our interest in their presumed role in innate immune responses in the snail hosts, we also performed an analysis of α3-FucT gene expression during *in vitro* miracidium-to-primary sporocyst development.

## Materials and Methods

### Isolation and Cultivation of *S. mansoni* Larva

#### Ethics statement

Research protocols involving mice, including routine maintenance and care, were reviewed and approved by the Institutional Animal Care and Use Committee (IACUC) at the University of Wisconsin-Madison under assurance no. A3368-01.

Adult and larval *S. mansoni* (NMRI strain) were obtained and axenically cultivated as previously described by Yoshino and Laursen [22]. Briefly, adults and eggs were harvested from the hepatic portal veins and livers, respectively, of infected mice 7–8 weeks post-exposure to infective cercariae. Livers were homogenized to release the trapped eggs, and miracidial hatching was triggered by incubation in artificial pond water [23]. Miracidia were either used immediately or induced to transform by cultivation at 26°C in Chernin’s Balanced Salt Solution (CBSS; 47.9 mM NaCl/2.0 mM KCl/0.5 mM Na_2_HPO_4_/1.8 mM MgSO_4_·7 H_2_O/3.6 mM CaCl_2_·2 H_2_O/0.6 mM NaHCO_3_; [24]) containing glucose and trehalose (1 g/L each) as well as penicillin and streptomycin (CBSS^+^). Within 24 h of cultivation, most miracidia had fully transformed to primary sporocysts. In this study, sporocyst cultures were maintained for 2 and 10 days before material extraction. For 10-day cultivations, the CBSS^+^ culture medium was changed on days 2 and 7.

### FucT Gene Identification

The amino acid sequences of previously characterized α2-, α3-, α6-, and protein O-FucTs of *S. mansoni*, *Homo sapiens*, *Drosophila melanogaster* and *Caenorhabditis elegans*, as well as the unique dual-function β3-galactosyltransferase/α2-FucT PgtA (also called FucB) of *Dictyostelium discoideum*, were downloaded from Reference Sequence (RefSeq) and GenBank online databases at the National Center for Biotechnology Information (NCBI) (accession numbers in Table S5). These sequences were used as queries in a genome-wide basic local alignment search tool [25] screen of genomic scaffolds and predicted genes in the *Schistosoma mansoni* database (SchistoDB).

### Primer Design

FucT oligonucleotide primers used in reverse transcriptase (RT)-PCR, rapid amplification of cDNA ends (RACE), and real-time quantitative (q)PCR reactions were designed using Vector NTI Advance 11.0 software (Invitrogen, Eugene, OR, USA) and the Integrated DNA Technologies (IDT) SciTools suite (www.idtdna.com/scitools/scitools.aspx) based on available SchistoDB-derived genomic sequence information and original data obtained by this study. Custom DNA oligonucleotides were purchased from IDT (Coralville, IA, USA). Primer sequences used in this study are provided in Tables S1, S2, S3, S4.

### Reverse Transcriptase-PCR and Rapid Amplification of cDNA Ends for FucT Transcript Sequencing

Unless otherwise stated, all kits and reagents were used according to the manufacturers’ recommendations. All protocols involving thermal cycling were executed on a Mastercycler® ep Thermal Cycler (Eppendorf North America, Hauppauge, NY, USA). Miracidia, 2-day *in vitro*-cultivated primary sporocysts, and mixed-sex adult worms were washed five times with artificial pond water, CBSS, and mammalian phosphate-buffered saline (pH 7.4), respectively, and total RNA was extracted using TRIzol® Reagent (Invitrogen). Genomic DNA contamination was removed from raw RNA extracts by TURBO™ DNase treatment (Applied Biosystems, Foster City, CA, USA), and the DNA-free RNA was converted to RT-PCR-ready cDNA using the SuperScript® III First-Strand Synthesis System (Invitrogen). Reverse transcriptase-PCR reactions (25 µL/rxn) contained GoTaq® amplification reagents (Promega, Madison, WI, USA), with reaction mixtures generally comprising 2.5 U GoTaq® Flexi DNA Polymerase, 1X Green GoTaq® Flexi Reaction Buffer, 400 nM each forward and reverse gene-specific primers (Table S1), 1.6 mM dNTP mix (400 µM each), 1.5 mM MgCl_2_, and 75–350 ng RNA input-equivalents RT-PCR-ready cDNA. The thermal profile was as follows: initial denaturation at 94°C/3 min; 40 cycles of 94°C/30 sec, 56–60°C/30 sec and 72°C/3 min; and final extension at 72°C/10 min. Some reactions required further optimization of cDNA input, annealing temperature, and cycle duration. Amplification products were fractionated by 1% agarose gel electrophoresis, and ethidium bromide-stained bands were excised and purified using a QIAquick Gel Extraction Kit (Qiagen, Germantown, MD, USA). Amplicons were inserted into pCR®4-TOPO® vector (TOPO® TA Cloning® Kit for Sequencing, Invitrogen) and transformed into One Shot® TOP10 Chemically Competent *E. coli*, which were then incubated overnight at 37°C on LB (1.0% tryptone/0.5% yeast extract/1.0% NaCl) agar (1.5%) containing 50 µg/mL kanamycin. Positive transformants were picked and grown overnight at 37°C in LB broth containing 100 µg/mL ampicillin, and plasmids were isolated using a QIAprep Spin Miniprep Kit (Qiagen). To verify the presence of an appropriate insert, plasmids were restriction-digested with EcoRI endonuclease (Promega, Madison, WI), and restriction fragments were analyzed by electrophoretic fractionation and ultraviolet transillumination. Insert-bearing plasmids were used as templates for dideoxy sequencing (BigDye Terminator v3.1; Applied Biosystems), and reaction products were purified using Agencourt® CleanSEQ® magnetic beads (Beckman Coulter, Brea, CA, USA). Following cleanup, insert sequences were read by the DNA Sequence Laboratory at the University of Wisconsin Biotechnology Center (Madison, WI, USA) using a 3730×l Automated DNA Sequencer (Applied Biosystems).

Following RT-PCR confirmation of gene transcription, TRIzol®-derived DNA-free total parasite RNA was converted to 5′ and 3′ RACE-ready cDNAs using a SMART™/SMARTer™ RACE cDNA Amplification Kit (Clontech, Mountain View, CA, USA), and cDNA ends were PCR-amplified using the Advantage® 2 PCR Kit (Clontech) with 200 nM gene-specific primers (Table S2), 240 nM universal primer mix (RACE kit component), and 20–100 ng RNA input-equivalents of RACE-ready cDNA (50 µL/rxn total volume). The thermal profile for RACE PCR reactions included initial denaturation at 94°C/3 min, 25–30 cycles of 94°C/30 sec, 58–62°C/30 sec and 72°C/3 min, and final extension at 72°C/10 min. Further optimization was required in some cases. Often, nested PCR was performed using the above thermal profile and recipe but with 200 nM nested gene-specific and universal primers and 2.5 µL diluted “outer” PCR reaction (1/50 dilution with Tricine-EDTA Buffer; Clontech). Amplification products were isolated, cloned, and sequenced as described above.

Reverse transcriptase-PCR and RACE sequence data were assembled and edited using Vector NTI Advance 11.0 software. Complete coding sequences (CDSs) were verified by RT-PCR amplification and subsequent sequencing (as above) using primers designed to encompass the full open reading frames (ORFs) (Table S3).

### Phylogenetic Analysis of *S. mansoni* FucT Genes

Amino acid sequences representing the known diversity of α2-, α3-, α6-, and protein O-FucTs from *Dictyostelium, Caenorhabditis*, *Drosophila*, *Danio*, *Mus*, and humans were compiled with our data from *S. mansoni* (Table S5). Alignments were generated using default settings in MUSCLE v3.6 [26], with manual correction in Mesquite [27]. An initial neighbor-joining tree was constructed using FastTree v2.0.1 [28] with a Jukes-Cantor+CAT model to serve as a guide tree for Bayesian phylogenetic inference. Analyses were then performed using mixed amino-acid models within MrBayes v3.1.2 [29] with two parallel runs of four Markov Chain Monte Carlo (MCMC) chains, each for five million generations, with subsampling every 100^th^ generation. Two independent replicates were conducted to determine that analyses were not trapped at local optima. Convergence of the MCMC chains was explored graphically using the online program AWTY [30], in addition to assessing stationarity of molecular evolutionary parameters by effective sample sizes >400 in Tracer v.1.5 [31]. Trees prior to stationarity were burned-in, and the remaining were used to assess posterior probabilities for nodal support. The bifunctional β3-galactosyltransferase/α2-FucT PgtA of *Dictyostelium discoideum* was used as an outgroup to facilitate inferences regarding FucT evolution.

Three predicted FucTs from *Schistosoma japonicum* (GenBank accession/SchistoDB annotation numbers CAX72936.1, CAX73054.1, and Sjp_0036210), in addition to the above data set, were used to construct a second phylogeny. FucT amino acid sequences were aligned, and a maximum-likelihood (ML) tree was inferred using the RAxML v7.3.4 [32] program, employing a general time-reversible (GTR) model of nucleotide substitution with Γ-distributed rate variation among sites. Statistical support for individual nodes within the best-scoring tree was estimated using the rapid bootstrap algorithm (1,000 replications) in RAxML.

### Real-time Quantitative PCR Analysis of α3-FucT mRNA Expression in Miracidia and Primary Sporocysts

Real-time qPCR was performed according to recommendations by Applied Biosystems (http://www.appliedbiosystems.com/absite/us/en/home/applications-technologies/real-time-pcr/), including strict criteria for qPCR primer design, validation, and optimization. Relative transcript abundance in miracidia and primary sporocysts was assessed using the comparative C_T_ (ΔΔC_T_) method, in which an endogenous calibrator gene is used to normalize the C_T_ values for a gene of interest. To identify appropriate calibrators for this study, the *Schistosoma mansoni* Serial Analysis of Gene Expression (SAGE) Database [33] was screened for genes whose transcript abundances are stable between miracidia and primary sporocysts (R-value <4; [34]). Based on the available SAGE data, ATP synthase f chain (herein termed “*ATPsf*”; SAGE tag 195 corresponding to Smp_140480 at SchistoDB) and the *GroES* chaperonin (SAGE tag 132 corresponding to Smp_097380) were selected. The compatibility of calibrator and α3-FucT primers under normal reaction conditions was assessed by plotting ΔC_T_ at various dilutions of cDNA input and determining the slope of the resultant line; primer efficiencies were deemed compatible if the absolute value of the slope was less than 0.1. Primers used in this study for qPCR are listed in Table S4.

Miracidia and *in vitro*-cultivated primary sporocysts were washed five times with artificial pond water and CBSS, respectively, and total RNA was extracted using TRIzol® Reagent. The raw RNA was decontaminated by TURBO™ DNase treatment and quantitatively converted to first-strand cDNA using the Superscript™III-First-Strand Synthesis System. Real-time qPCR reactions (50 µL/rxn) were prepared in triplicate, comprising 1X SYBR Green PCR Master Mix (Applied Biosystems), 20 ng RNA input-equivalents parasite cDNA, and 200 nM each forward and reverse gene-specific primers. Reactions were run on an ABI 7300 Real-Time PCR System (Applied Biosystems) with the following cycle profile: initial denaturation at 95°C/10 min followed by 40 cycles of 95°C/15 sec and 60°C/1 min. PCR product accumulation was monitored in real time, and amplification fidelity was assessed by post-cycling thermal dissociation and electrophoretic fractionation.

To best assess α3-FucT expression by the ΔΔC_T_ method, the geometric mean of *ATPsf* and *GroES* C_T_ values was used to normalize α3-FucT C_T_ such that ΔC_T_ = C_T-FucT_ − C_T-GeoMean(*ATPsf*, *GroES*)_. Heteroscedastic Student’s T-tests were employed to compare α3-FucT expression in miracidia versus primary sporocysts across three independent biological replicates, with significance set at *p*≤0.05.

## Results and Discussion

### Composition, Genomic Organization, and Splicing of Schistosome FucT Genes

An exhaustive homology search of the *Schistosoma mansoni* database (SchistoDB; [35]) in conjunction with comprehensive sequence analyses generated a non-redundant list of 15 genes with predicted roles in α3-, α6- and protein O-fucosylation (see Table 1 for genes and corresponding SchistoDB annotations). Seven genes were classified as putatively involved in α3-fucosylation (herein termed *FucT*s *A*,*B*,*C*,*D*,*E*,*F*,*G*), six in α6-fucosylation (*FucT*s *H*,*I*,*J*,*K*,*L*,*M*), and two in protein O-fucosylation (*POFucT*s *A*,*B*). No genes with a predicted role in α2-fucosylation were identified. Of the 15 FucT homologs described here, only *FucTA* had been previously cloned and characterized [20]. Homology-based searches failed to detect any sequences in the SchistoDB corresponding to *FucT-VII*, the only other FucT homolog reported from *S. mansoni* [19]. Notably, subsequent attempts to clone the *FucT-VII* CDS from miracidia, primary sporocysts, and adult worms were unsuccessful despite the use of numerous primer sets and various amplification parameters (see discussion below).

**Table 1:** Summary of FucT genomic organization in *Schistosoma mansoni*.

Gene	Gene ID a	Scaffold ID a	Approx. size (bp)	No. of exons	ORF length (nt) b	Prot. length (aa) b
*FucTA*	Smp_148850	Smp_scaff000127	>16,057	8	1,278	426
*FucTB*	Smp_099090	Smp_scaff000622	≥7,903	6	1,248	416
*FucTC*	Smp_154410	Smp_scaff000171	≥24,925	10	1,389	463
*FucTD*	Smp_054300	Smp_scaff000144	≥9,546	7	1,194	398
*FucTE*	Smp_137740	Smp_scaff000060	>21,917	8	1,284	428
*FucTF*	Smp_137730	Smp_scaff000060	>10,507	7	1,302	434
*FucTG*	Smp_129750	Smp_scaff000024	≥12,334	10	–	–
*FucTH*	Smp_175120	Smp_scaff000594	>38,746	11	1,797	599
*FucTI*	–	Smp_scaff000066	–	10	1,776	592
*FucTJ*	Smp_138730	Smp_scaff000066	>15,639	10	1,776	592
*FucTK*	Smp_138750	Smp_scaff000066	>37,696	10	1,737	579
*FucTL*	Smp_030650	Smp_scaff000066	>24,336	10	1,764	588
*FucTM*	–	Smp_scaff000066	–	10	1,776	592
*POFucTA*	Smp_065240	Smp_scaff000199	≥9,121	9	1,251	417
*POFucTB*	Smp_131810	Smp_scaff000033	>27,578	12	1,650	550

a Smp gene and scaffold IDs refer to nomenclature in the SchistoDB [35].

b ORF and protein sizes are provided for the main/major transcripts. Alternative splicing may alter ORF length and protein coding.

Previously, Fitzpatrick et al. [21] used a similar bioinformatics approach (Pfam searches) to identify predicted α3-FucTs for inclusion in a microarray analysis of gene expression in *S. mansoni*. Their approach generated eight unique contigs/sequences (seven corresponding to present *FucT*s *A*-*G*, plus one more), but no further sequence analyses were conducted to validate them as complete α3-FucT-coding genes. The eighth putative FucT (SchistoDB annotation Smp_194990) was not incorporated in their microarray analysis and its transcription was not confirmed. In the present study, Smp_194990 was excluded from downstream analyses because it is ostensibly incomplete, comprising four exons that constitute just 804 nt of a potential FucT CDS. While it is possible the remaining coding segments reside within a genomic sequencing gap, the nearest gap is ∼32 kb downstream and represents a distance much longer than the introns of other schistosome FucTs. Altogether, 13 such “gene fragments” (all α3-FucT-like) were discarded as probable pseudogenes and not analyzed further.

To confirm FucT gene transcription in *S. mansoni* and obtain full-length CDSs, transcript sequences were RT-PCR- and RACE-amplified using cDNA derived from miracidia, primary sporocysts, and adults. Complete sequences were submitted to the NCBI GenBank (see Table S5 for accession numbers). In most cases, *in silico* translation of gene transcripts yielded a single prevailing ORF. However, translation of *FucTG* revealed two tandemly situated ORFs corresponding to different segments of a single α3-FucT protein, indicating premature termination of translation. Indeed, sequence analyses determined that exon 8 of *FucTG* encodes a premature termination codon (PTC) while exon 9 forces a downstream frameshift (see Figure S2), both resulting in the omission of a major segment of the FucT catalytic domain. Moreover, *in silico* analyses of FucTG membrane topology suggested the absence of an N-terminal transmembrane domain (TMD), a structural element observed in every known eukaryotic α3-FucT [36]. Altogether, our data suggest that *FucTG* is a pseudogene.

Nucleotide sequence data were mapped onto the corresponding SchistoDB-derived genomic scaffolds to examine FucT gene organization (Table 1; Figure S1). With the exceptions of *FucTE* and *FucTF*, which are tandemly situated on scaffold Smp_scaff000060, α3-FucT genes occur on separate genomic scaffolds. In contrast, schistosome α6-FucTs are distributed between just two scaffolds, with *FucT*s *I*-*M* arrayed on Smp_scaff000066 and *FucTH* on Smp_scaff000594. Scaffold Smp_scaff000066 features SchistoDB-annotated gene predictions corresponding to α6-*FucT*s *J*–*L* (Table 1), but no obvious matches for *FucTI* and *FucTM*. The scaffold does include a fourth SchistoDB annotation for a putative FucT gene (Smp_138740), however all attempts by RT-PCR and RACE to confirm its expression failed. Notably, sequence comparisons show that *FucTI* and *FucTM* are almost identical to Smp_138740, differing in their CDS regions by 12 and 15 nt, respectively (>99% identity in both comparisons). Moreover, the CDSs of *FucT*s *I*, *J*, and *M* are very similar, differing from one another by just 11 (*I* vs. *J*), 23 (*I* vs. *M*), and 20 (*J* vs. *M*) nt. Given the high similarity among these sequences (both exonic and intronic) and the close proximity of Smp_138740 to *FucTJ* within the genome, it is conceivable that *FucT*s *I*, *J*, and *M* were incorrectly consolidated during contig assembly into two tandem genes. Indeed, the 5′ half of the *FucTI* mRNA transcript (including the untranslated region) is identical to that of *FucTJ* while its 3′ end is almost identical to the 3′-coding segments of upstream Smp_138740.

Also apparent by genomic overlay, schistosome FucTs are invariably multiexonic, with CDSs spanning 5–8 exons among the α3-FucTs, 9 or 11 exons in the α6-FucTs, and 9–10 exons in the protein O-FucTs (Figure S1). The observation that *FucTA* is multiexonic contradicts a previous assertion by Trottein et al. [20] that its CDS is fully encoded by a single exon. Similar multiexonic organizations have been observed for FucTs of other invertebrates and plants [36]–[38], as well as a subset of vertebrate genes, including members of the *FUT10*/*11* superfamily, *FUT*s *7*–*8*, and protein O-FucTs *POFUT1* and *POFUT2* [37], [39]–[41]. In contrast, vertebrate *FUT*s *1*–*6* and *FUT9* are all monoexonic [36], [42]. Notably, genomic overlay further revealed that the proportioning of schistosome α3- and α6-FucT CDSs among their multiple exons is roughly maintained (i.e., exon-exon junctions are well conserved within gene families). Similar conservation of ORF-exon architecture has been observed among vertebrate α3-FucTs (e.g., human *FUT*s *3*–*6*/*9*; [36]) and for the protein O-FucTs across a broad diversity of invertebrate and vertebrate taxa [37].

The significance of a multiexonic gene organization is perhaps most apparent in the context of alternative splicing. Variations in mRNA splicing were observed for all genes except α3-*FucTD* and α6-*FucTM* (Figure S2). It should be noted that due to the extreme similarity between the 5′ regions of *FucTI* and *FucTJ*, it is unclear whether the observed splicing occurs in one or both genes. Also, many of these observations were derived from RT-PCR and RACE experiments that targeted specific sections of each transcript and not the entire ORF. For this reason, the relationships among alternative splice events (i.e., whether events are co-dependent in the formation of particular isoforms) are largely unknown. All modes of alternative splicing were observed: exon skipping (e.g., *FucTF*), intron retention (e.g., *FucTA*), mutual exclusion (e.g., exons 1 and 2 of *FucTH*), and use of alternate splice donor and acceptor sites (e.g., *FucTH*). An *in silico* analysis to define the consequences of alternative splicing determined that most variant splice events would alter protein coding by introducing a PTC, forcing a downstream frameshift, effecting an in-frame deletion or addition, or omitting the prototypical start or stop codon. Few alternative events are predicted to leave the prototypical ORF unchanged. Additional studies are necessary to determine the true biochemical effects of such variations.

In general, alternative splicing is the primary mechanism by which organisms generate greater mRNA structural complexity, thus expanding proteome diversity, facilitating post-transcriptional gene regulation (e.g., introduction of a PTC that results in nonsense-mediated decay), and enhancing untranslated region variability (affecting cis-regulatory elements that control translation efficiency, stability, and localization) (reviewed by [43]). In terms of fucosylation in *Schistosoma*, alternative splicing among the FucT genes might generate additional FucT diversity (possibly modifying acceptor specificity, localization, or catalytic efficiency) or cause transcripts to be targeted for nonsense-mediated decay, perhaps effecting a reduction in FucT protein expression. Alternative splicing also has complex roles at the cellular and organismal levels in the modulation of physiological activities during development and differentiation and in response to environmental stresses [43]. Indeed, the developmental regulation of alternative splicing in *S. mansoni* has been well documented. For example, Ram et al. [44] observed cercariae-specific intron retention in heat-shock transcription factor (*HSF*) transcripts that introduces a PTC and prohibits HSF translation, thus inhibiting downstream expression of the molecular chaperone heat-shock protein 70 (*HSP70*). In adults, the impeding intronic sequences are removed, allowing functional HSF protein to be synthesized and ultimately promoting *HSP70* expression. In another study, DeMarco et al. [45] demonstrated by semi-quantitative RT-PCR that several protein-coding variants of the schistosome transcriptional cofactor *CA150* are expressed in different ratios between male and female adults. In the present study, the results of similar RT-PCR experiments suggest that at least a subset of the schistosome α3-FucT genes may also be differentially spliced among miracidia, primary sporocysts, and adults (unpublished data). Ultimately, the regulated expression of particular isoforms may have a role in generating the observed stage- and gender-specific patterns of fucosylation in *S. mansoni*.

### *In silico* Characterization of Schistosome FucT Proteins

To provide support for the putative functions of the 15 schistosome FucT homologs, their predicted amino acid sequences were compared within their respective gene families and against previously well characterized FucTs, and proteins were examined for the presence of key FucT-associated primary sequence elements. By definition, Golgi-resident α2-, α3-, and α6-FucTs are type-II transmembrane proteins featuring a single TMD, which is flanked by a short cytoplasmic N-terminal tail and a lumenal C-terminus that comprises a globular catalytic domain and a flexible hypervariable stem [46].

**Figure 1:**
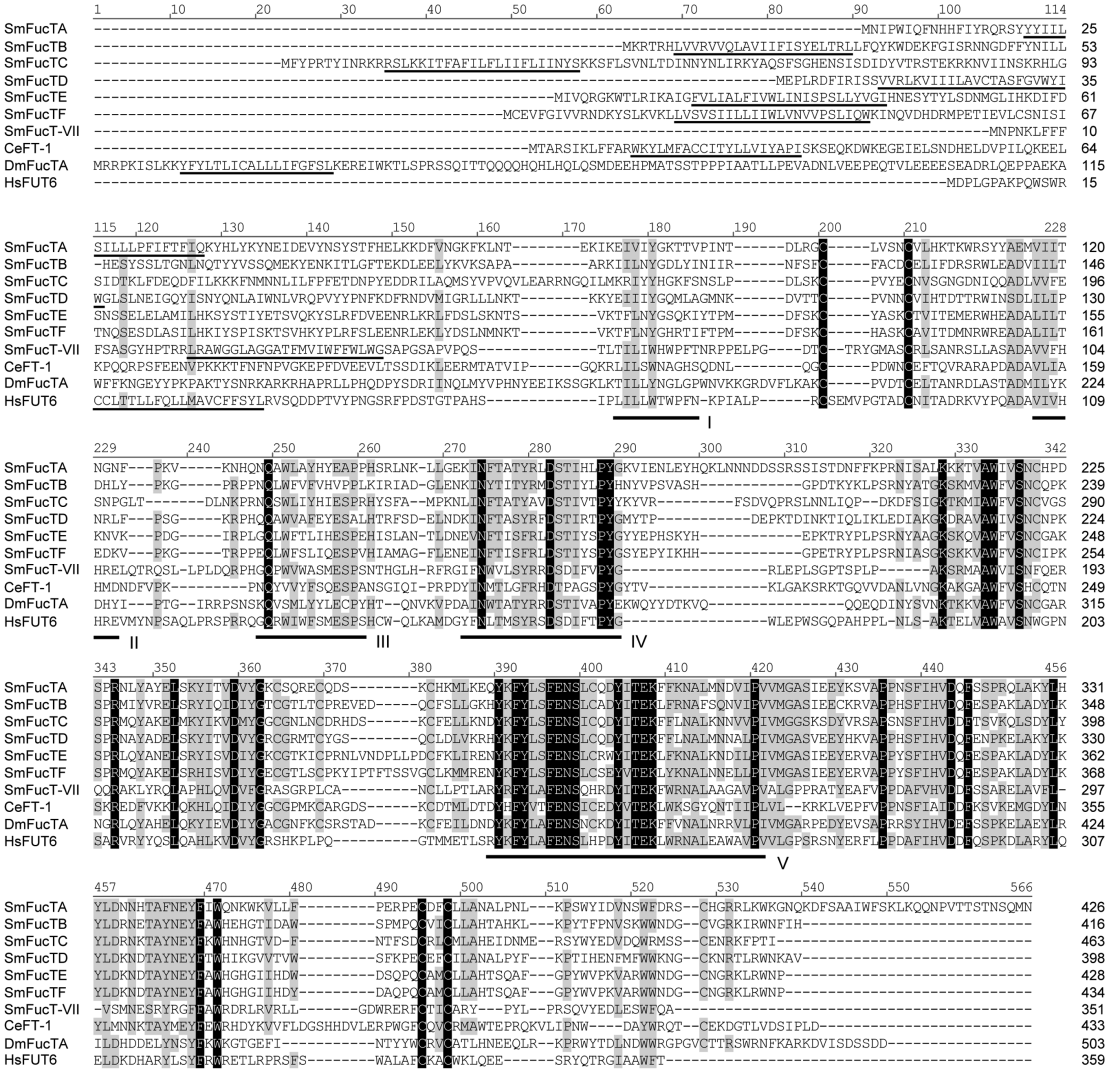
Amino acid alignment of α3-fucosyltransferases (α3-FucTs). Schistosome α3-FucTs are compared to FT-1, FucTA, and FUT6 of *Caenorhabditis elegans*, *Drosophila melanogaster* and humans, respectively (NCBI accession numbers in Table S5). Alignment position is indicated above each block, and sequence length is reported to the right of each line. Positions exhibiting greater than 70% conservation are highlighted in gray, and identities are shown in black. An N-terminal TMD (underlined) was identified for each FucT using TMHMM 2.0 and/or the TMpred online server (settings: min = 14/max = 23). The positions of five α3-FucT-specific motifs (motifs I-V; [41]) are indicated. Motifs IV and V were previously described in human α3-FucTs as motifs I and II [36], [51], and present motifs I and II were formerly termed motif III and “acceptor-binding motif”, respectively [49], [50]. Vector NTI Advance 11.0 software alignment settings: BLOSUM45 matrix with Cys and Trp weights adjusted to 99, gap opening penalty = 12, gap extension penalty = 0.1, gap separation penalty range = 0, no residue-specific or hydrophobic residue gaps; manual editing was necessary to fully align conserved motifs.

Alignment of the schistosome putative α3-FucTs (including FucT-VII; primary sequences obtained by *in silico* translation) against homologs of *Caenorhabditis*, *Drosophila*, and humans revealed the presence of an N-terminal hypervariable region and a well-conserved C-terminal constant domain in each protein (Figure 1). Additionally, the alignment identified five α3-FucT-specific motifs (I-V; [41]) that have roles in protein folding and catalytic activity (motif I; [47], [48]), acceptor specificity (motif II; [49], [50]), and GDP-L-fucose binding (motifs IV and V; [36], [51]). Motifs III-V are well conserved for all sequences, but motifs I and II differ both between taxa and within the schistosome gene family. These variations likely reflect differences in acceptor utilization, evidenced by interspecific variations in their glycomes [52]–[54]. Analyses of transmembrane topology using TMHMM 2.0 [55] and the TMpred server [56] suggest that all schistosome α3-FucTs (excluding FucTG) are type-II transmembrane proteins featuring a single N-terminal TMD. In all cases, the TMD is offset from the C-terminal catalytic domain by a hypervariable stem region, which varies in length among family members (e.g., 13 aa in FucT-VII versus 118 in FucTC). Stem length in any Golgi-resident glycosyltransferase is thought to contribute to acceptor specificity by positioning the catalytic domain at a particular distance from the Golgi membrane and providing constraints on the sterics of the glycosylation reaction [57]. Overall, the schistosome α3-FucT gene family shares ∼7–10% identity with *Caenorhabditis*, *Drosophila* and human homologs, while alignment of the schistosome α3-FucTs alone indicates ∼10% identity (∼17% if FucT-VII is omitted). Pairwise comparisons revealed 28.9–68.7% identity among schistosome genes.

**Figure 2:**
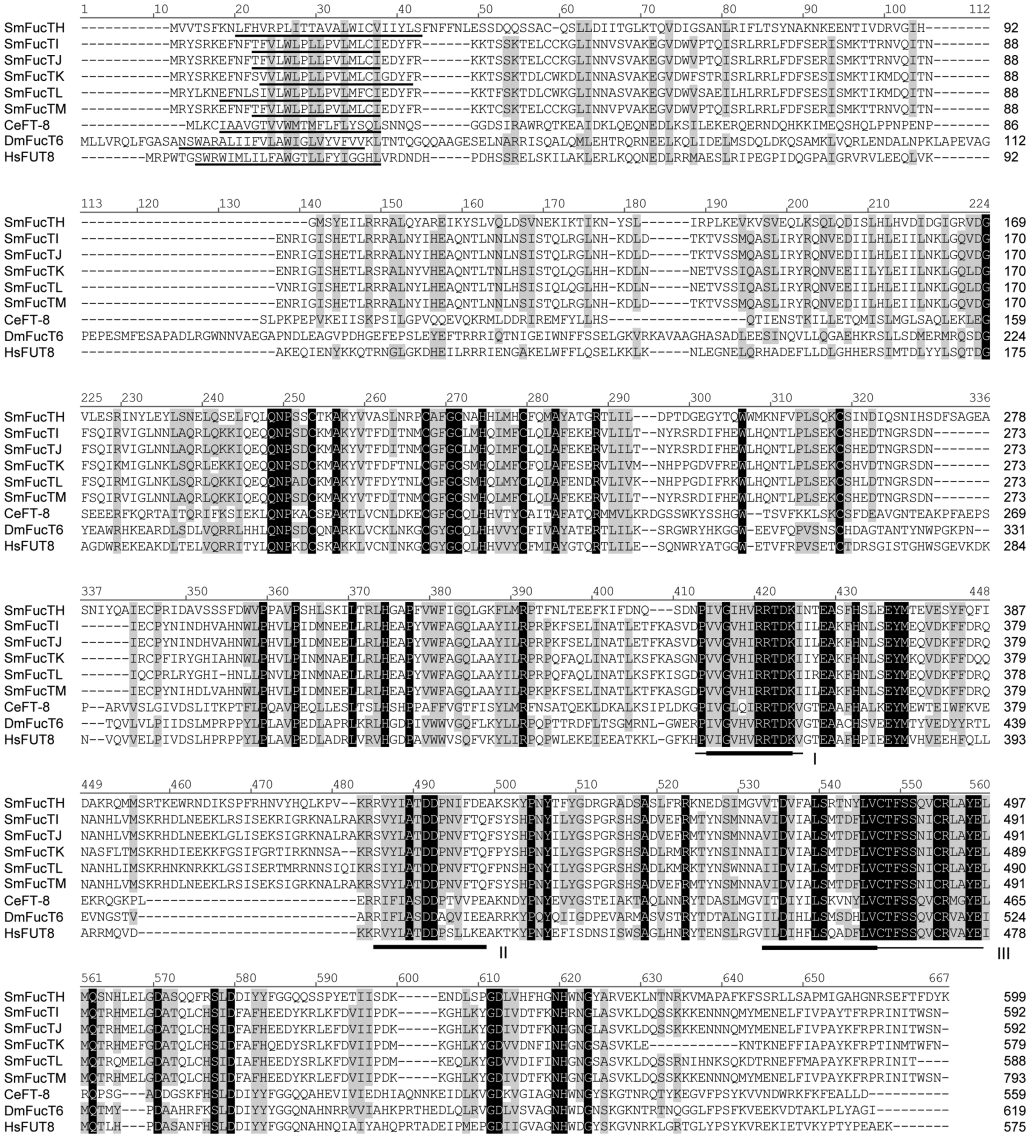
Amino acid alignment of α6-fucosyltransferases (α6-FucTs). Schistosome α6-FucTs are compared to FT-8, FucT6, and FUT8 of *Caenorhabditis elegans*, *Drosophila melanogaster* and humans, respectively (NCBI accession numbers in Table S5). Alignment position is indicated above each block, and sequence length is reported at the end of each line. Positions of identity and positions exhibiting at least 70% conservation are highlighted in black and gray, respectively. An N-terminal TMD (underlined) was identified for each FucT using TMHMM 2.0 and/or the TMpred online server (settings: min = 14/max = 23). The positions of three hydrophobic cluster analysis-derived motifs (I-III), which are well conserved among α2-, α6- and protein O-FucTs [36], [51], [58], are indicated below the alignment blocks. The reported lengths of motifs I and III differ between previous studies, which is reflected in the thickness of the indicator line (thick line, [36]; thin line, [58]). Vector NTI Advance 11.0 software alignment settings: BLOSUM45 matrix with Cys and Trp weights adjusted to 99, gap opening penalty = 12, gap extension penalty = 0.1, gap separation penalty range = 0, no residue-specific or hydrophobic residue gaps.

Similar observations were made regarding the schistosome α6-FucTs, which also comprise N-terminal variable and C-terminal constant regions (Figure 2). Alignment of these proteins against invertebrate and vertebrate homologs revealed the presence of three motifs that are well conserved across the α2-, α6- and protein O-FucT gene families [36], [51], [58]. Importantly, motif I residues Arg-420 and Arg-421, which are thought to be essential for GDP-L-fucose binding [59], are maintained in all schistosome α6-FucTs. Analyses of transmembrane topology strongly indicate that schistosome α6-FucTs also have a single N-terminal TMD with a type II orientation. Overall, schistosome α6-FucTs are ∼13–15% identical to *Caenorhabditis*, *Drosophila* and human homologs, while ∼27% sequence identity exists within the schistosome gene family (73% if FucTH is excluded from the analysis). Intrafamilial pairwise alignments demonstrated as much as 99% identity (FucTI vs. FucTJ).

**Figure 3:**
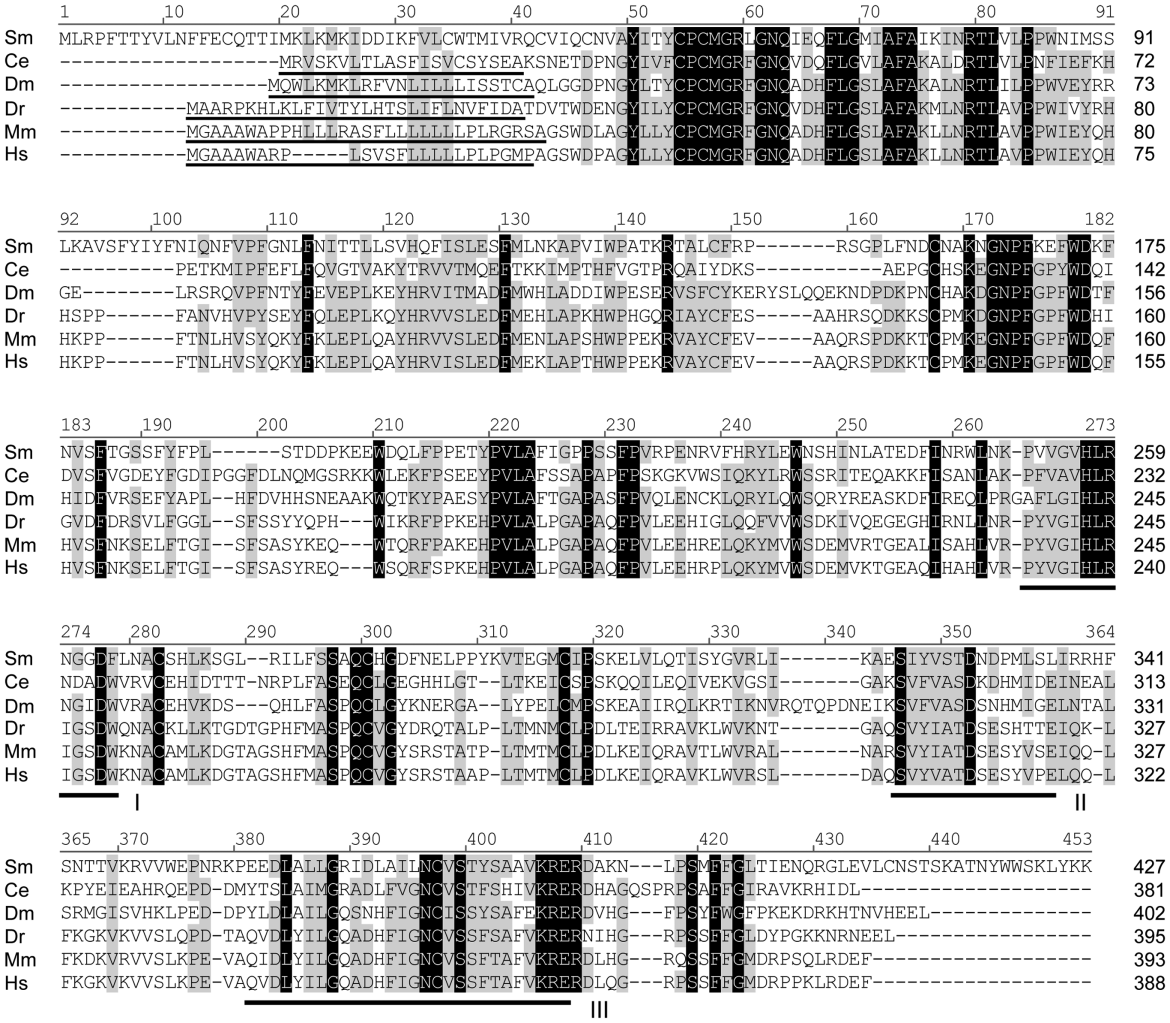
Amino acid alignment of protein O-fucosyltransferase 1 (O-FucT1) sequences. Schistosome POFucTA is compared to O-FucT1 sequences of *Caenorhabditis elegans* (Ce), *Drosophila melanogaster* (Dm), *Danio rerio* (Dr), *Mus musculus* (Mm), and humans (Hs) (NCBI accession numbers in Table S5). Alignment position is indicated above each block, and sequence length is reported to the right of each line. Positions of identity and positions exhibiting at least 70% conservation are highlighted in black and gray, respectively. The positions of three hydrophobic cluster analysis-derived motifs (I-III), which are shared features among α2-, α6- and protein O-FucTs [36], [51], [58], are indicated below the alignment blocks. Amino-terminal signal peptides (underlined) were identified using SMART and/or Phobius online tools. Vector NTI Advance 11.0 software alignment settings: BLOSUM45 matrix, gap opening penalty = 12, gap extension penalty = 0.1, gap separation penalty range = 0, no residue-specific or hydrophobic residue gaps.

**Figure 4:**
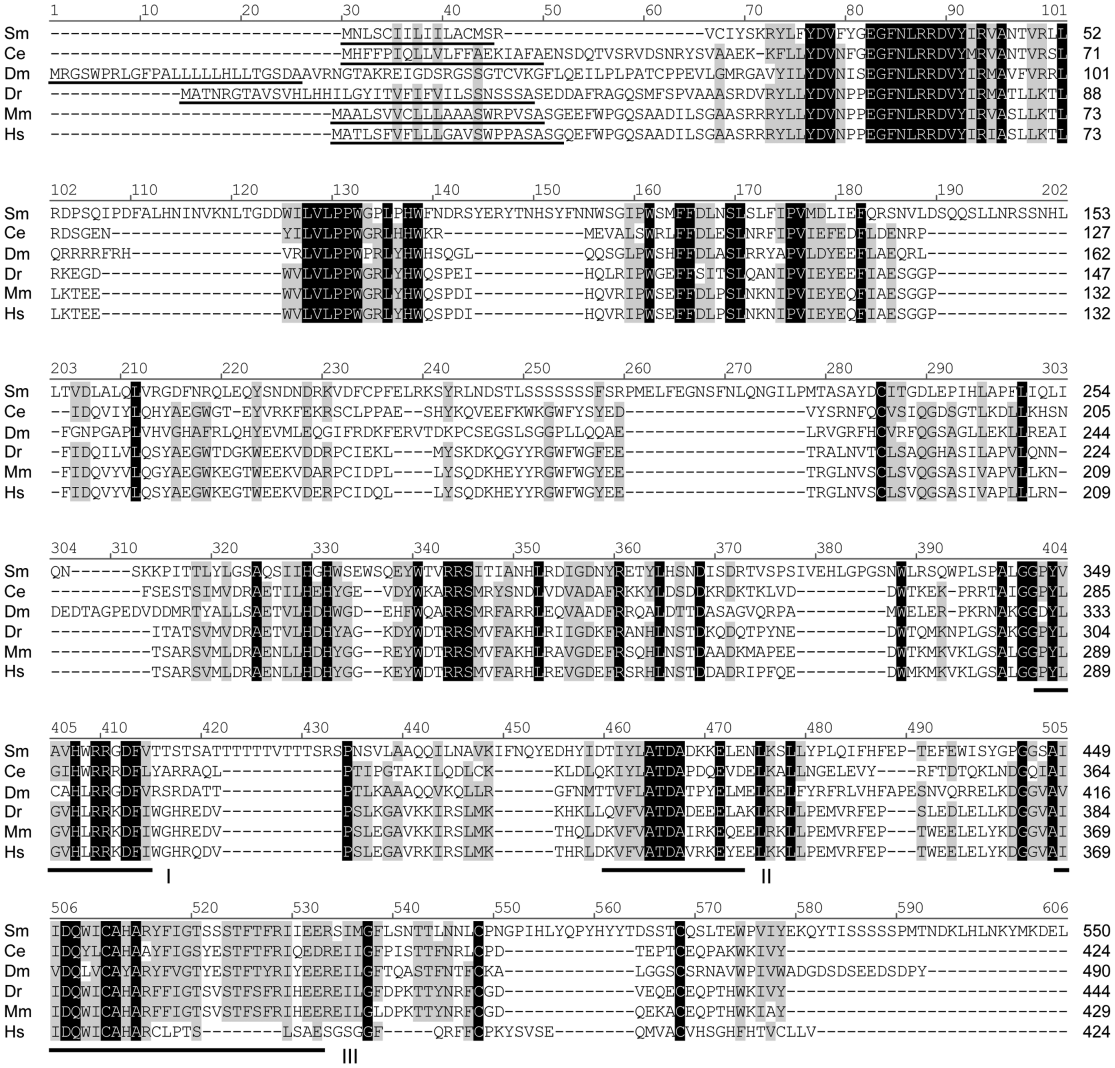
Amino acid alignment of protein O-fucosyltransferase 2 (O-FucT2) sequences. Schistosome POFucTB is compared to O-FucT2 sequences of *Caenorhabditis elegans* (Ce), *Drosophila melanogaster* (Dm), *Danio rerio* (Dr), *Mus musculus* (Mm), and humans (Hs) (NCBI accession numbers in Table S5). Alignment position is indicated above each block, and sequence length is reported to the right of each line. Identical and conserved (>70%) positions are highlighted in black and gray, respectively. The positions of three hydrophobic cluster analysis-derived motifs (I-III), which are shared features among members of the α2−/α6−/O-FucT superfamily [36], [51], [58], are indicated below the alignment blocks. An N-terminal signal peptide (underlined) was identified in all sequences using the SMART and/or Phobius servers. Note, the human O-FucT2 RefSeq protein used in the present analysis lacks motif III, however another version of human POFUT2 that includes this motif is available at NCBI (GenBank accession number AAH64623.1). Vector NTI Advance 11.0 software alignment settings: BLOSUM45 matrix, gap opening penalty = 12, gap extension penalty = 0.1, gap separation penalty range = 0, no residue-specific or hydrophobic residue gaps.

Unlike α2-, α3- and α6-FucTs, protein O-FucTs (comprising distinct O-FucT1 and O-FucT2 gene families) are predominantly ER-localized soluble proteins that transfer L-fucose to serine and threonine residues of epidermal growth factor- and thrombospondin-type repeats [60]–[62]. Amino-terminal signal peptides, which initially target proteins to the ER, have been described for both O-FucT1 and O-FucT2 proteins [37], [61], [62], and C-terminal ER-retention/retrieval signals have been observed in most O-FucT1s studied to date [37], [63], [64]. Alignments of schistosome POFucTA and POFucTB against O-FucT1 and O-FucT2 orthologs, respectively, show that both proteins are well conserved in *S. mansoni* (∼25–33% identity in pairwise alignments; Figures 3 and 4) and that both proteins contain three key motifs that are putatively involved in GDP-L-fucose binding [36], [51], [58]. A search for N-terminal signal peptides was conducted using the Simple Modular Architecture Research Tool (SMART; [65]) and the Phobius transmembrane topology and signal peptide prediction server [66], and signal peptides were successfully identified in all sequences except schistosome POFucTA. Consistent with the expectation that protein O-FucTs are soluble, the Phobius output indicated the absence of a TMD in all cases. Protein sequences were also examined for a C-terminal ER-retention/retrieval signal such as the soluble protein motif KDEL or similar tetrapeptides, e.g., HEEL and RDEF of *Drosophila*, *Mus*, and human orthologs, or the membrane protein dibasic motifs KKxx and RRx [61], [67], [68]). Unlike most of its O-FucT1 orthologs, schistosome POFucTA lacks a KDEL-like tetrapeptide. Strikingly, the C-terminus of POFucTA terminates with a KK tandem repeat that is reminiscent of a membrane protein-associated dibasic ER sorting signal. However, lysine residues are inaptly spaced from the terminus and are therefore unlikely to participate in retrograde transport. It is unclear given the absence of both an N-terminal signal sequence and a retention/retrieval signal if or how POFucTA is initially targeted to and later retained in the ER, where it is predicted to function in protein O-fucosylation. In contrast to POFucTA, schistosome POFucTB features an N-terminal signal sequence and a classical C-terminal KDEL ER-retention/retrieval tetrapeptide. Importantly, this is the first report of an ER-retention/retrieval signal in a protein O-FucT2 to date.

### Phylogenetic Analysis of Schistosome FucTs

**Figure 5:**
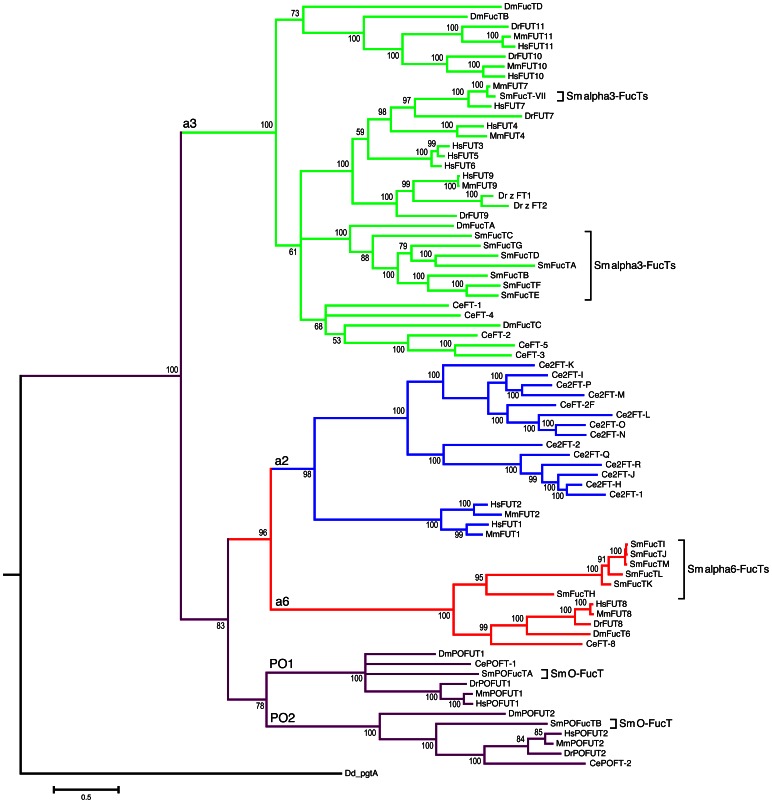
Phylogenetic tree of fucosyltransferases (FucTs). In addition to the FucTs of *Schistosoma mansoni* (Sm), the α2-, α3-, α6- and protein O-FucTs from *Caenorhabditis elegans* (Ce), *Drosophila melanogaster* (Dm), *Danio rerio* (Dr), *Mus musculus* (Mm), and humans (Hs) were selected to represent the diversity of known, well-characterized FucTs (NCBI accession numbers and references in Table S5), and used to construct a molecular phylogeny rooted on the bifunctional β3-galactosyltransferase/α2-FucT PgtA of *Dictyostelium discoideum* (Dd). Posterior probabilities ≥50% are indicated at each node, and genetic divergence (substitutions per site) is represented by the scale bar.

A phylogenetic analysis was conducted to examine the relationship between the schistosome FucT homologs and 62 previously characterized α2-, α3-, α6-, and protein O-FucTs of *Dictyostelium*, *Caenorhabditis*, *Drosophila*, *Danio*, *Mus* and humans (Figure 5; see Table S5 for NCBI accession numbers). The resultant tree, which is rooted on a bifunctional β3-galactosyltransferase/α2-FucT from *Dictyostelium* (PgtA; [69], [70]), resolves the data into five major clades that correspond to the α2-, α3-, α6-, O-FucT1 and O-FucT2 gene families, and it supports previous work demonstrating that gene duplication and functional divergence among FucTs is a relatively ancient event [58], with members of each gene family being represented across invertebrate and vertebrate taxa.

The formation of a superclade comprising the α2-, α6-, and protein O-FucT lineages is consistent with observations by Martinez-Duncker et al. [58] that these distinct functional groups constitute a single superfamily of FucTs. Indeed, three well-conserved motifs, which are thought to have a role in GDP-L-fucose binding, are shared across the α2-, α6- and protein O-FucT families [36], [51], [58]. Analogous motifs are also conserved among the α3-FucTs (motifs IV and V; [36]), however the relationship of these motifs to motifs I-III of the α2−/α6−/O-FucT superfamily is unclear. The topology of the α2−/α6−/O-FucT superfamily clade suggests two main lineages, the α2−/α6- and protein O-FucT lines, that were derived by duplication of an ancestral gene followed by functional divergence. For the O-FucT lineage this meant the loss of an N-terminal TMD and alteration of acceptor specificity (oligosaccharides to proteins) and subcellular localization (Golgi to ER). More recently, the ancestral α2−/α6- and protein O-FucT genes underwent a second round of duplication and functional divergence to form the modern α2-, α6-, O-FucT1, and O-FucT2 gene families. The schistosome FucTs clustered predictably within the α2−/α6−/O-FucT superfamily, with *Schistosoma* being represented in all but the α2-FucT clade. POFucTA and POFucTB are clear orthologs of the O-FucT1 and O-FucT2 gene families, respectively, while the schistosome α6-FucT homologs constitute their own monophyletic group within the α6- lineage.

Consistent with observations by Roos et al. [71] and Mollicone et al. [41], the present phylogeny predicts two distinct lineages within the α3-FucT clade, one comprising the FUT10/11 gene superfamily and the other encompassing human FUT*s* 3–7/FUT9 and their orthologs, as well as distinct groups from *Schistosoma* and *Caenorhabditis*. With the exception of FucT-VII, schistosome α3-FucTs comprise a monophyletic group. The close proximity of schistosome FucT-VII to murine Fut7 and its relative distance from the main schistosome lineage supports previous claims that *FucT-VII* is an artifact of *in vitro* contamination or a product of a recent horizontal gene transfer from mouse to schistosome [36]. Indeed, type-VII FucTs, including schistosome FucT-VII, are associated with α3-fucosylation of sialylated Lewis-type oligosaccharide acceptors [19], [39], which have never been observed in *S. mansoni*. Oriol et al. [36] favored the notion of lateral gene transfer because Marques et al. [19] reportedly detected *FucT-VII* mRNA expression in snail-derived larvae and hamster-reared adults. However, given failed attempts in the present study to PCR-amplify *FucT-VII* and locate the relevant gene sequence in the SchistoDB database, it is perhaps more likely that *FucT-VII* was derived in the previous work by *in vitro* contamination.

In contrast to the schistosome α3-FucT gene family, *Drosophila* α3-FucTs exhibit a polyphyletic distribution, with two of these genes appearing more closely related to the FUT10/11 superfamily and two others divided between the schistosome and *Caenorhabditis* lineages. Mollicone et al. [41] concluded that *Drosophila* FUT10/11-like α3-FucTs share a common ancestor with the present FUT10/11 superfamily. Because schistosomes apparently lack a FUT10/11-like gene, the origin of the FUT10/11 superfamily is likely more recent than the separation of schistosomes from the insect and vertebrate lineages.

Interestingly, the layering of gene organization data onto the present tree is highly informative regarding α3-FucT gene evolution. Invertebrate α3-FucTs and FUT10/11 superfamily genes are all multiexonic, while vertebrate *FUT*s *3*–*7* and *FUT9* feature a much-simplified genomic organization (encoded by just one exon; two in *FUT7*). All of the bi- and monoexonic vertebrate genes form a monophyletic clade, suggesting that *FUT*s *3*–*7*/*9* were derived by successive duplication after the retrotransposition of a single ancestral gene. The introduction of a single intron into *FUT7* is perhaps a more recent event in the evolution of the vertebrate α3-FucTs. Furthermore, because this simplified gene organization is conserved across vertebrate taxa (*Danio*, *Mus*, and humans) and not among invertebrates, it can be concluded that retrotransposition must have occurred after the separation of vertebrate and invertebrate lineages but early in vertebrate evolution. Notably, Marques et al. [19] observed that schistosome *FucT-VII* is monoexonic, which is consistent with its monophyletic relationship with *Mus* and human *FUT7* genes in the present phylogenetic tree and further supports hypotheses that *FucT-VII* is a product of *in vitro* contamination or horizontal gene transfer from mice.

Monophyly within the schistosome α3- and α6-FucT gene families, in conjunction with the tandem organization of some genes in the genome and their conserved ORF-exon architecture, suggests that multiplicity among the α3- and α6-FucTs likely derived by successive segment duplications (rather than retrotranspositions) and that such duplications, especially among the α6-FucTs, are relatively recent events. Indeed, Silva et al. [72], using a phylogenomic approach to identify lineage-specific gene duplications, concluded that expansion of the FucT gene family was among the most significant to have occurred in *S. mansoni*. In general, the downstream consequences for duplicated genes include nonfunctionalization (collection of degenerative mutations), neofunctionalization (attainment of a new function), and subfunctionalization (partitioning of the original function between duplicate genes) (reviewed by Hurles [73]). All three outcomes are evident within the α3-FucT gene family. The *FucTG* pseudogene is a likely example of nonfunctionalization, while neofunctionalization and subfunctionalization could be evidenced by gene-specific variations in acceptor specificity (as observed in *Caenorhabditis*; [38]) and developmentally regulated gene expression (described below; also see [21]), respectively. Furthermore, given the observed lack of α2-FucT homologs in the schistosome genome and the expression of a unique Fucα1-2Fuc linkage [74], it is possible that one (or more) of the α3−/α6- paralogs has neofunctionalized to add fucose in an α2 linkage instead of (or in addition to) the predicted α3/α6 linkage. Indeed, previous studies have demonstrated that some forms of human FUT3 have the ability to generate α2 linkages in addition to the usual α3 and α4 linkages [75], [76].

Finally, a similar phylogenetic analysis incorporating three unverified homologs from a second human-infective schistosome, *S. japonicum* (predicted genes herein referenced by GenBank accession/SchistoDB annotation numbers CAX72936.1, CAX73054.1, and Sjp_0036210), generated a topologically concordant tree in which the putative *S. japonicum* FucTs form monophyletic groups with those of *S. mansoni* (Figure S3). Moreover, the phylogeny identifies *CAX72936.1* and *Sjp_0036210* as orthologs of *FucTH* and *FucTB*, respectively, and indicates that *CAX73054.1* shares a single ancestral node with *FucT*s *I*-*M*. Interestingly, the topology within the schistosome α6-clade implies a significant expansion of the α6-FucT gene family in *S. mansoni* following the evolutionary separation of *S. mansoni* and *S. japonicum*. However, the complete repertoire of *S. japonicum* FucT genes has yet to be resolved and future investigations may identify additional α6- (and α3-) orthologs.

### Real-time Quantitative PCR Analysis of α3-FucT mRNA Expression in Miracidia and Primary Sporocysts

**Figure 6:**
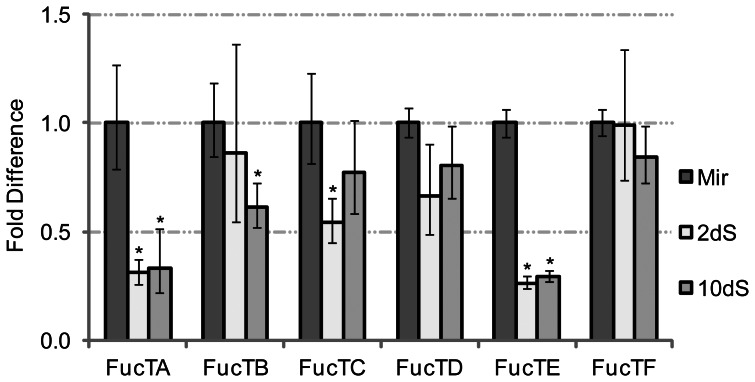
Alpha3-fucosyltransferase (α3-FucT) gene transcription in larvae of ***Schistosoma mansoni.*** Real-time qPCR was used to examine steady-state levels of α3-FucT transcription in miracidia (Mir) and 2- and 10-day *in vitro*-cultivated primary sporocysts (2 dS and 10 dS, respectively). Transcript abundance in primary sporocysts was assessed relative to miracidia, which was arbitrarily set at 1. Data represent the average of three independent biological replicates, and asterisks (*) indicate significantly altered gene transcription (*p*<0.05).

Given recent data demonstrating the abundant expression of fucosylated glycotopes in snail-associated schistosome larvae [10] and their predicted immunomodulatory roles in snail hosts, α3-FucT transcript expression was assayed in miracidia and 2- and 10-day *in vitro*-cultivated primary sporocysts (Figure 6). Real-time qPCR data indicate that *FucTA* and *FucTE* transcript abundance in larvae decreases as much as 74% during the miracidium-to-primary sporocyst transformation and remains low during *in vitro* cultivation. In contrast, expression of *FucTB* appears to stay high throughout larval transformation and only declines with extended cultivation (∼40% reduction), while *FucTC* expression exhibits the opposite trend, initially dropping ∼50% and then returning to near miracidial levels by day 10 in culture. No significant variations in transcript abundance were observed for *FucTD* and *FucTF* in these experiments. During the miracidium-to-primary sporocyst transformation, miracidia shed their ciliated epidermal plates and the associated glycocalyx and form a syncytial tegument [77]. Peterson et al. [10] showed that miracidial epidermal plates are dominated by fucosylated glycotopes, many of which are lost with the epidermal plates. Perhaps *FucTA* and *FucTE* are highly expressed in the epidermal plates and significant portions of their transcript populations are released with the plates. Similarly, the *FucTC* transcripts might also be lost during epidermal plate shedding, but transcription may increase in primary sporocysts during extended larval cultivation.

Previously, Fitzpatrick et al. [21] conducted a microarray analysis of FucT gene expression in all stages of the schistosome lifecycle, including miracidia and 2-day *in vitro*-cultivated primary sporocysts. Their results demonstrated that transcript levels for *FucT*s *D*-*F* are relatively high in the intramolluscan larval stages, with *FucTD* equally expressed between larvae, *FucTE* declining ∼50% during transformation, and *FucTF* peaking in primary sporocysts. Transcript levels for *FucTA* and *FucTB* were relatively low in both miracidia and primary sporocysts. *FucTC* expression was not assessed. In all cases, FucT transcript abundance is elevated in adults, and for some genes (e.g., *FucTB*) array data indicate significant differences in expression between males and females. Unfortunately, the above observations in intramolluscan larvae are incongruous with the results of the present study. Disparities may be due to methodological differences between studies, however Fitzpatrick et al. [24] appropriately validated the array data for *FucTA* and *FucTD* using methods similar to those employed here (real-time qPCR but with normalization against alpha tubulin). Regardless of which dataset more accurately describes α3-FucT gene expression in *S. mansoni*, both suggest that expression is developmentally regulated, which possibly contributes to the observed stage- and gender-specific expression of fucosylated glycotopes.

### Conclusions

The present study used a genome-wide homology-based bioinformatics approach to identify and *in silico* characterize the complete repertoire of FucT homologs that presumably contribute to fucosylation, especially for the synthesis of terminal glycans, in *S. mansoni*. Our search yielded 15 complete genes, including seven α3-FucTs, six α6-FucTs and two protein O-FucTs. Why schistosomes encode such a large number of FucT homologs remains unclear, however it is thought that such duplicative expansions are an adaptive response to the parasitic lifestyle and imply important roles for these genes in schistosome development and immunobiology [72]. Notably, this level of redundancy also exists in the non-parasitic nematode *Caenorhabditis* (see Figure 5; Table S5), which Oriol et al. [36] attributes to the evolutionary selection of fucosylation over sialylation as a means of terminating glycosylation. Indeed, sialic acid is absent from the *Caenorhabditis* glycome [78]. Likewise, sialic acid does not occur among the glycans of *Schistosoma* [79]–[81], indicating that fucosylation is the only means of terminal modification in schistosome glycosylation.

The observed redundancy in the α6-FucT gene family alone is quite interesting given that most invertebrate and vertebrate species examined to date feature just one such gene. The singular known function of these genes is to add fucose in an α6 linkage to the proximal GlcNAc of the N-glycan chitobiose core [18]. It is possible that some of the schistosome α6- paralogs have neofunctionalized or are functionally compartmentalized (as with the α3-FucTs of humans [82]), with FucTs featuring distinct expression patterns (i.e., tissue and stage specificity) and subtle variations in substrate utilization. Future studies should assess the tissue localization and stage-specificity of α6-FucT expression across the schistosome lifecycle.

Strikingly, despite the prominence of a unique Fucα1-2Fuc linkage in schistosome glycoconjugates, no α2-FucT homologs were identified in the present study. Because genomic sequence assembly for *S. mansoni* is not yet complete, with gaps still scattered throughout the genome [83], it is possible that α2-FucT homologs are encoded but were not detected due to insufficient sequence information. However, given the uniqueness of the Fucα1-2Fuc linkage and the apparent lack of Fucα1-2Gal linkages in *S. mansoni*, the absence of a conventional α2-FucT is not confounding. Alternatively, as described above, one of the predicted α3- or α6-FucTs may have neofunctionalized to create α2 linkages or a novel enzyme may exist, unrelated to currently recognized α2-FucTs, that serves this function. Future investigations should re-examine the schistosome genome for α2-FucT sequences as new data are generated, as well as functionally test the present FucTs for α2-fucosylation activity.

While protein O-FucTs are not directly involved in fucosylated glycotope expression, O-FucT1 and O-FucT2 play essential roles in diverse developmental and physiological processes. It is likely that these enzymes play many of the same roles in *S. mansoni*. A cursory search of the NCBI RefSeq database yielded schistosome homologs of O-FucT substrate-coding genes, including notch (accession number XM_002574857.1; [84]), ADAMTS5 peptidase (XM_002571852.1, [85]), properdin (XM_002580039.1; [86]), and f-spondin (XM_002581166.1; [86]). Future research should examine the role of schistosome O-FucTs in modifying these substrates and determine their significance in the context of schistosome development and immunobiology.

Unfortunately, attempts in this study to biochemically define the schistosome FucT homologs were unsuccessful, and assertions regarding their putative functions are based solely on our *in silico* analyses and are thus inherently speculative. Additional biochemical studies clearly are required to demonstrate FucT activities and link them to glycotope expression. However, the present work has provided an essential framework that will serve to inform and motivate future investigations exploring the role of fucosylation in schistosome development and immunobiology. Furthermore, this study highlighted several possible gene targets for the development of novel anti-schistosomal vaccines and chemotherapeutics.

## Supporting Information

Figure S1 **Diagrammatic depiction of fucosyltransferase (FucT) genomic organization in** ***Schistosoma mansoni*** **.** The mRNA transcript sequences of schistosome FucTs were mapped onto SchistoDB-derived genomic scaffolds. Exons (boxes, numbered below) and introns (connecting lines) are drawn to scale (bar = 1000 nt) with FucT-coding elements (segments of the prototypical ORF), including exons and a subset of retained introns, depicted as black boxes and non-coding exons depicted as gray boxes. Caret marks indicate gaps in the genomic sequence, and dotted lines represent introns of unknown length (spacing instead based on closely related FucTs).
(TIF)

Figure S2 **Diagrammatic depiction of fucosyltransferase (FucT) gene alternative splicing in** ***Schistosoma mansoni*** **.** Alternative splicing, including exon skipping, intron retention, mutual exclusivity among exons and use of alternate splice donor and acceptor sites, was observed during transcript sequencing. Bent connectors indicate splicing between exons (boxes, numbered above), with solid lines representing splicing in the main/major full-length FucT-coding transcripts and dotted lines representing alternative splice events. Exons are drawn to scale (bar = 500 nt) and spacing of exons is arbitrary. Interexonic boxes represent retained introns (estimated lengths in parentheses), with solid outlines signifying retention in the main/major transcript and dotted lines indicating retention in other isoforms. Positions of the prototypical start and stop codons (AUG and UAA/UGA/UAG, respectively) are shown. Colors convey the *in silico* consequences of splicing: black, conservation of the prototypical ORF; red, introduction of a premature termination codon; orange, induction of a downstream frameshift; green, in-frame deletion/addition; blue, omission of the prototypical start/stop codon. Exon 4 of *FucTE* is tandemly duplicated (exon 5, white box); it is unknown if splice isoforms show preference for one copy versus the other.
(TIF)

Figure S3 **Phylogeny of fucosyltransferases (FucTs), including FucT homologs of** ***Schistosoma japonicum*** **.** A phylogenetic tree was constructed using the maximum likelihood method and a GTR+Γ substitution model implemented in RAxML v.7.3.4. The FucTs of *Schistosoma mansoni* (Sm; marked by bars on right) as well as the α2-, α3-, and α6- and protein O-FucTs from *Caenorhabditis elegans* (Ce), *Drosophila melanogaster* (Dm), *Danio rerio* (Dr), *Mus musculus* (Mm), and humans (Hs) were selected to represent the known FucT diversity (see for accession numbers). Additionally, three predicted FucTs of *Schistosoma japonicum* (labeled with GenBank accession/SchistoDB annotation numbers CAX72936.1, CAX73054.1, and Sjp_0036210) were included. The tree was rooted on the bifunctional β3-galactosyltransferase/α2-FucT PgtA of *Dictyostelium discoideum* (Dd). Numbers above or below branches indicate bootstrap support (%) estimated from 1,000 resamplings of the sequence data; bootstrap values ≤50% are not shown. Genetic divergence (substitutions per site) is represented by the scale bar.
(TIFF)

Table S1 Primers used for reverse transcriptase-PCR confirmation of fucosyltransferase gene transcription.
(DOCX)

Table S2 Primers used for 5′ and 3′ rapid amplification of cDNA ends (RACE) of fucosyltransferase gene transcripts.
(DOCX)

Table S3 Primers used for reverse transcriptase-PCR amplification of fucosyltransferase complete coding sequences.
(DOCX)

Table S4 Primers used for quantitative PCR analyses of α3-fucosyltransferase gene transcript expression.
(DOCX)

Table S5 NCBI RefSeq/GenBank accession numbers (number.version) of referenced fucosyltransferase genes.
(DOCX)
