# Supplementary figures and images for: In Silico Analysis of the Fucosylation-Associated Genome of the Human Blood Fluke Schistosoma mansoni: Cloning and Characterization of the Fucosyltransferase Multigene Family
Source: PLoS One. 2013 May 16;8(5):e63299. doi: 10.1371/journal.pone.0063299 (PMC3655985; doi:10.1371/journal.pone.0063299)

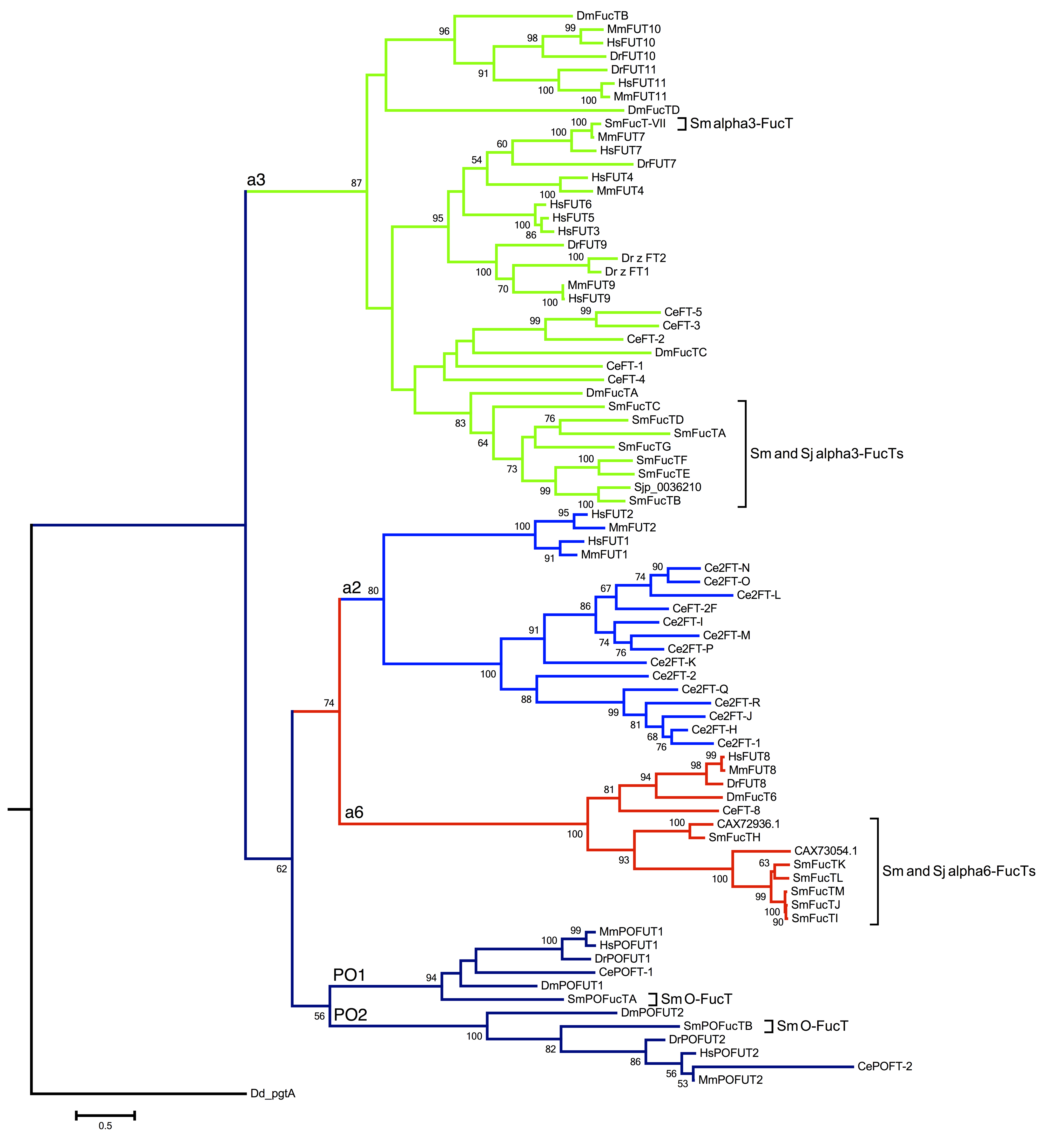

Supplement: Figure S3 — Phylogeny of fucosyltransferases (FucTs), including FucT homologs of Schistosoma japonicum . A phylogenetic tree was constructed using the maximum likelihood method and a GTR+Γ substitution model implemented in RAxML v.7.3.4. The FucTs of Schistosoma mansoni (Sm; marked by bars on right) as well as the α2-, α3-, and α6- and protein O-FucTs from Caenorhabditis elegans (Ce), Drosophila melanogaster (Dm), Danio rerio (Dr), Mus musculus (Mm), and humans (Hs) were selected to represent the known FucT diversity (see for accession numbers). Additionally, three predicted FucTs of Schistosoma japonicum (labeled with GenBank accession/SchistoDB annotation numbers CAX72936.1, CAX73054.1, and Sjp_0036210) were included. The tree was rooted on the bifunctional β3-galactosyltransferase/α2-FucT PgtA of Dictyostelium discoideum (Dd). Numbers above or below branches indicate bootstrap support (%) estimated from 1,000 resamplings of the sequence data; bootstrap values ≤50% are not shown. Genetic divergence (substitutions per site) is represented by the scale bar. (TIFF) [file pone.0063299.s003.tiff]
