# Supplementary material for: In Silico Analysis of the Fucosylation-Associated Genome of the Human Blood Fluke Schistosoma mansoni: Cloning and Characterization of the Fucosyltransferase Multigene Family
Source: PLoS One. 2013 May 16;8(5):e63299. doi: 10.1371/journal.pone.0063299 (PMC3655985; doi:10.1371/journal.pone.0063299)
Supplement: Table S1 — Primers used for reverse transcriptase-PCR confirmation of fucosyltransferase gene transcription. (DOCX) [file pone.0063299.s004.docx]

**Supplementary** **Table S1**. **Primers used for reverse transcriptase-PCR confirmation of fucosyltransferase gene transcription**

| **Gene** | **Forward** | **Reverse** |
| --- | --- | --- |
| FucTB | 5′-CCCTAGAGAAGTCGAAGATCAATGT-3′ | 5′-TTCCGACCAACACAACCATC-3′ |
| FucTC | 5′-GCACAGTCATTCAGTGGACATG-3′ | 5′-TCAGCCAAGATTGATTCCTCG-3′ |
| FucTD | 5′-CGTCCACATCAACAGGCTTG-3′ | 5′-TGGCCAGTTCTTTCGGATTC-3′ |
| FucTE | 5′-TCATTGCCCTTTTCATTGTATGG-3′ | 5′-TCGTTATCCAGTGTGTTTGCCA-3′ |
| FucTF | 5′-TGAAAGTCAAATTGTTGGTCAGTG-3′ | 5′-GGACATGATAACGTACCACACTC-3′ |
| FucTG | 5′-CCACATAATCAGGCATGGATTG-3′ | 5′-CCATTTGCCAACCAAGAATGA-3′ |
| FucTH | 5′-AATAGACCTTGTGCATTCGGTTGTAATGC-3′ | 5′-CTTGCATAACCATTCCAATGATTACCATG-3′ |
| FucTI | - | - |
| FucTJ | 5′-GAAGAATTATTGCGTTTACATGAGGCACCC-3′ | 5′-TCCCATGATTTTTGAAGGTATCCACTATG-3′ |
| FucTK | 5′-AGCGCGTTTTAATAGTGATGAATCACCCG-3′ | 5′-TGAACCAAGTCATGTTTATTGTTGGTCG-3′ |
| FucTL | 5′-ACTTTGACCAATTTACACAGCATTTCTATC-3′ | 5′-TGTGACCATATCGAAGACGAGGACATTG-3′ |
| FucTM | - | - |
| POFucTA | 5′-AACCTACTGTCCTTGCATGGGTCGGCTC-3′ | 5′-CCACCAGTAGTTGGTAGCTTTACTTGTGG-3′ |
| POFucTB | 5′-CCACCTTGGGGTCCACTTCCTCATTGG-3′ | 5′-GCCGAACCACCTGGGCCATAAGATATCCAC-3′ |
