# Supplementary material for: In Silico Analysis of the Fucosylation-Associated Genome of the Human Blood Fluke Schistosoma mansoni: Cloning and Characterization of the Fucosyltransferase Multigene Family
Source: PLoS One. 2013 May 16;8(5):e63299. doi: 10.1371/journal.pone.0063299 (PMC3655985; doi:10.1371/journal.pone.0063299)
Supplement: Table S2 — Primers used for 5′ and 3′ rapid amplification of cDNA ends (RACE) of fucosyltransferase gene transcripts. (DOCX) [file pone.0063299.s005.docx]

**Supplementary** **Table S2**. **Primers used for 5′ and 3′ rapid amplification of cDNA ends (RACE) of fucosyltransferase gene transcripts**

| **Gene** | **Forward (3′ RACE)** | **Reverse (5′ RACE)** |
| --- | --- | --- |
| FucTB | 5′-AGCGTGTTGCTCCTCCTCAT-3′ | 5′-ACACATTGAGGCATAGGTGACCAAGCAT-3′ |
|  | 5′-GATGGTTGTGTTGGTCGGAA-3′ | 5′-CCGACCAACACAACCATCGTTCCAC-3′ |
| FucTC | 5′-GAGACAATATCCAGCAAGCCGATTTAGTCG-3′ | 5′-CGACTAAATCGGCTTGCTGGATATTGTCTC-3′ |
|  | 5′-CCATACGAGGATGATAGGATATTGGCGCAA-3′ | 5′-CCATACGAGGATGATAGGATATTGGCGCAA-3′ |
|  | 5′-GCACAGTCATTCAGTGGACATGAGAACTCA-3′ | 5′-TGAGTTCTCATGTCCACTGAATGACTGTGC-3′ |
| FucTD | 5′-TGCCTCAGTTGAAGAGTATCACAAGGTTGC-3′ | 5′-GCAACCTTGTGATACTCTTCAACTGAGGCA-3′ |
|  | 5′-TGGTCGATGTGGTCGGATGACCTGCTACGG-3′ | 5′-CCGTAGCAGGTCATCCGACCACATCGACCA-3′ |
|  | 5′-GGATGACCTGCTACGGTTCACAGTGTCTTG-3′ | 5′-CAAGACACTGTGAACCGTAGCAGGTCATCC-3′ |
| FucTE | 5′-CGATTCAAACTCTTCAGAGTTAGAACTGGC-3′ | 5′-GCCAGTTCTAACTCTGAAGAGTTTGAATCG-3′ |
|  | 5′-GATGGAATTCGGCCACTTGGACAATTATGG-3′ | 5′-CCATAATTGTCCAAGTGGCCGAATTCCATC-3′ |
| FucTF | 5′-CAAGAGTCGCCTGTACATATTGCTATGGC-3′ | 5′-TGTACGATGCCCATAGTTGAGAAACGTC-3′ |
|  | 5′-TGGTGAGTGTGGTACGTTATCATGTCCA-3′ | 5′-CCATGGTGCTTTATGTATGGCTCATAGG-3′ |
| FucTG | 5′-TCCACATAATCAGGCATGGATTGGATTTAG-3′ | 5′-CTAATCCAATCCATGCCTGATTATGTGGA-3′ |
|  | 5′-AGAAACCACGGAACAATCAAGTCTGAGACG-3′ | 5′-CGTCTCAGACTTGATTGTTCCGTGGTTTCT-3′ |
|  | 5′-TGATGAATATTTTGCATGGCATGTTCACGG-3′ | 5′-CCGTGAACATGCCATGCAAAATATTCATCA-3′ |
|  | 5′-GTTGATATGTATGGAGGTTGTGGAGGTTCG-3′ | 5′-CGAACCTCCACAACCTCCATACATATCAAC-3′ |
| FucTH | 5′-AATAGACCTTGTGCATTCGGTTGTAATGC-3′ | 5′-CTTGCATAACCATTCCAATGATTACCATG-3′ |
|  | 5′-TGGGGAGGGATACACACAATGGTGGATG-3′ | 5′-CATCAATTCATAAGCTAATCGACAAACCTG-3′ |
| FucTI | 5′-GAAGAATTATTGCGTTTACATGAGGCACCC-3′ | 5′-TGTCAGTACGTCTGATATGCACACCAACT-3′ |
|  |  | 5′-TGCTAGTTGGCCAGCGAACCAAACATAG-3′ |
| FucTJ | 5′-GAAGAATTATTGCGTTTACATGAGGCACCC-3′ | 5′-TCCCATGATTTTTGAAGGTATCCACTATG-3′ |
|  | 5′-GCACCACCATCTTTTTTCCAGCGTCCC-3′ |  |
| FucTK | 5′-AGCGCGTTTTAATAGTGATGAATCACCCG-3′ | 5′-TGAACCAAGTCATGTTTATTGTTGGTCG-3′ |
|  | 5′-ATCCCATTCAGCTGATTTGAGGATGCGG-3′ | 5′-GGGTGATTCATCACTATTAAAACGCGCTC-3′ |
| FucTL | 5′-AGAAGAAGATTCAAGAGCAACAGAATCCTG-3′ | 5′-TGTGACCATATCGAAGACGAGGACATTG-3′ |
|  | 5′-CAGATAACATCCAATGTCCTCGTCTTCG-3′ | 5′-CTGTGAAAGCTTATTTAAGCCAATCATTCG-3′ |
| FucTM | - | 5′-GGCAACCAGTTATGTGCTACGAGATCATG-3′ |
| POFucTA | 5′-AACCTACTGTCCTTGCATGGGTCGGCTC-3′ | 5′-CCACCAGTAGTTGGTAGCTTTACTTGTGG-3′ |
|  | 5′-AAGCTCCCGTCATCTGGCCAGCAAC-3′ | 5′-AGGTTTTCGATTAGGTTCCCACACAACACG-3′ |
| POFucTB | 5′-CCACCTTGGGGTCCACTTCCTCATTGG-3′ | 5′-ATCTGGCATGTGCACAGATCCATTGATC-3′ |
|  | 5′-TAGCAAGACAACCATCCAGGGACGTTGGTG-3′ | 5′-GCCGAACCACCTGGGCCATAAGATATCCAC-3′ |
