# Supplementary material for: In Silico Analysis of the Fucosylation-Associated Genome of the Human Blood Fluke Schistosoma mansoni: Cloning and Characterization of the Fucosyltransferase Multigene Family
Source: PLoS One. 2013 May 16;8(5):e63299. doi: 10.1371/journal.pone.0063299 (PMC3655985; doi:10.1371/journal.pone.0063299)
Supplement: Table S3 — Primers used for reverse transcriptase-PCR amplification of fucosyltransferase complete coding sequences. (DOCX) [file pone.0063299.s006.docx]

**Supplementary** **Table S3**. **Primers used for reverse transcriptase-PCR amplification of fucosyltransferase complete coding sequences**

| **Gene** | **Forward** | **Reverse** |
| --- | --- | --- |
| FucTA | 5′-CATGGATACAATTTAATCACCA-3′ | 5′-TAATTCATTTGTGAATTGGTC-3′ |
| FucTB | 5′-TTGTGTCAATTACAAGGGATG-3′ | 5′-TAATGGATAAAATTCCAGCG-3′ |
| FucTC | 5′-ATCATCAACACGATCTTTGC-3′ | 5′-TAAATAGTCGGAAATTTACGA-3′ |
| FucTD | 5′-CACTATGGAACCTCTTAGAGACT-3′ | 5′-TAAACAGCTTTATTCCATCTTA-3′ |
| FucTE | 5′-ATGGGTTTAATATAGTTGGCA-3′ | 5′-ATGGATTCCAACGCAATTTC-3′ |
| FucTF | 5′-ATCCCACATTTCTGATGATCAATCC-3′ | 5′-CTTTCATGGATTCCAACGCAATTTCCG-3′ |
| FucTG | 5′-AGGGTTCCAGCGTTCAAGTG-3′ | 5′-CTGTCCAATGCTATCGAAGACGCATAG-3′ |
| FucTH | 5′-AAGTGGCTTGTGATTCCCACTGTG-3′ | 5′-AACACCATGAGTAGTGTGTACATACACG-3′ |
| FucTI | 5′-CAATAAGGACGCTAGACAGATCATACTGG-3′ | 5′-CGGCGCACAATTGAATCCAAAG-3′ |
| FucTJ | 5′-CAATAAGGACGCTAGACAGATCATACTGG-3′ | 5′-TAGGATAATGTCAGGCATCAAAAGGGAC-3′ |
| FucTK | 5′-GTTGAAGGCCAAAATACCAGATCATGAG-3′ | 5′-GAAGCTGGAAAAAAGATGGCGGTAT-3′ |
| FucTL | 5′-TTGAAGGCCAAAATACCAGATCATGAG-3′ | 5′-GTTCCTAACTATGTGTAGGCTGATGTAAG-3′ |
| FucTM | 5′-GATCATGCTGTTCGAACGTCATTAATTTG-3′ | 5′-CGGCGCACAATTGAATCCAAAG-3′ |
| POFucTA | 5′-TCTTGTCCTGTGGCATCTAGACGAG-3′ | 5′-GCAACTGTCAATCAGTCGTCTCAGAC-3′ |
| POFucTB | 5′-TTACGCGTAGCTATGTCGTTCCTG-3′ | 5′-CTTACTTACTTACGCCTGTTACCCCTC-3′ |
