# Supplementary material for: In Silico Analysis of the Fucosylation-Associated Genome of the Human Blood Fluke Schistosoma mansoni: Cloning and Characterization of the Fucosyltransferase Multigene Family
Source: PLoS One. 2013 May 16;8(5):e63299. doi: 10.1371/journal.pone.0063299 (PMC3655985; doi:10.1371/journal.pone.0063299)
Supplement: Table S4 — Primers used for quantitative PCR analyses of α3-fucosyltransferase gene transcript expression. (DOCX) [file pone.0063299.s007.docx]

**Supplementary** **Table S4**. **Primers used for quantitative PCR analyses of α3-fucosyltransferase gene transcript expression**

| **Gene** | **Forward** | **Reverse** |
| --- | --- | --- |
| FucTA | 5′-CTGTAACTACATCGACCAATTCAC-3′ | 5′-TGTTCACATCACAACTCCATCAC-3′ |
| FucTB | 5′-ACAGCAGATCCAGTTTTGTGTC-3′ | 5′-GAACTACCCGTACAACTAAATGC-3′ |
| FucTC | 5′-ATCATCAACACGATCTTTGCCG-3′ | 5′-GTACGTGGGTAAAACATCACAG-3′ |
| FucTD | 5′-TCACTATGGAACCTCTTAGAGAC-3′ | 5′-TACCAAACACCAAAGCTAGCAG-3′ |
| FucTE | 5′-GTAAATGATCCCTTGTTGCCTG-3′ | 5′-GCTTCTCAGTTATATACCATCTACAC-3′ |
| FucTF | 5′-CCAACTTTCACCTCATCAGTTG-3′ | 5′-CTTCTCTGTTACATATTCACTGCAC-3′ |
| ATPsf | 5′-GAATACAATGCACGAGTACATGG-3′ | 5′-CTACGTGAAAACCACTCACTG-3′ |
| GroES | 5′-AAGTCGTACTGGACGAAAATGAG-3′ | 5′-GGACTAGATCTAAAACACCGGTC-3′ |
