# Supplementary material for: In Silico Analysis of the Fucosylation-Associated Genome of the Human Blood Fluke Schistosoma mansoni: Cloning and Characterization of the Fucosyltransferase Multigene Family
Source: PLoS One. 2013 May 16;8(5):e63299. doi: 10.1371/journal.pone.0063299 (PMC3655985; doi:10.1371/journal.pone.0063299)
Supplement: Table S5 — NCBI RefSeq/GenBank accession numbers (number.version) of referenced fucosyltransferase genes. (DOCX) [file pone.0063299.s008.docx]

**Supplementary Table S5. NCBI RefSeq/GenBank accession numbers (number.version) of referenced fucosyltransferase genes**

| **Source organism (kingdom, phylum, class, order, family ^a^)** | **Gene name/ID ^b^** | **Phylogenetic tree symbol** | **Nt accession** | **Prot. accession** | **Function ^c,d^** | **References** |
| --- | --- | --- | --- | --- | --- | --- |
| *Homo sapiens* (Anamalia, Chordata, Mammalia, Primates, Hominidae) | **FUT1** | Hs_FUT1 | NM_000148.3 | NP_000139.1 | α2-FucT | 1, 2 |
|  | **FUT2** | Hs_FUT2 | NM_000511.5 | NP_000502.4 | " " | 3, 4 |
|  | **FUT3** | Hs_FUT3 | NM_000149.3 | NP_000140.1 | α3/4-FucT (dual) | 5-8 |
|  | **FUT4** | Hs_FUT4 | NM_002033.3 | NP_002024.1 | α3-FucT | 9 |
|  | **FUT5** | Hs_FUT5 | BC140905.1 | AAI40906.1 | α3/4-FucT (dual) | 10 |
|  | **FUT6** | Hs_FUT6 | NM_000150.2 | NP_000141.1 | α3-FucT | 11, 12 |
|  | **FUT7** | Hs_FUT7 | NM_004479.3 | NP_004470.1 | " " | 13 |
|  | **FUT9** | Hs_FUT9 | NM_006581.3 | NP_006572.2 | " " | 14 |
|  | **FUT10** | Hs_FUT10 | NM_032664.3 | NP_116053.3 | " " | 15-17 |
|  | **FUT11** | Hs_FUT11 | NM_173540.2 | NP_775811.2 | " " | " " |
|  | **FUT8** | Hs_FUT8 | NM_178155.2 | NP_835368.1 | α6-FucT (core) | 18- 20 |
|  | **POFUT1** | Hs_POFUT1 | NM_015352.1 | NP_056167.1 | O-FucT | 21, 22 |
|  | **POFUT2** | Hs_POFUT2 | NM_015227.4 | NP_056042.1 | " " | 23 |
| *Mus musculus* (Anamalia, Chordata, Mammalia, Rodentia, Muridae) | Fut1/MFUT-I | Mm_Fut1 | NM_008051.5 | NP_032077.2 | α2-FucT | 24-26 |
|  | Fut2/MFUT-II | Mm_Fut2 | NM_018876.3 | NP_061364.2 | " " | 25-28 |
|  | Fut4 | Mm_Fut4 | NM_010242.3 | NP_034372.1 | α3-FucT | 29, 30 |
|  | Fut7 | Mm_Fut7 | NM_013524.3 | NP_038552.1 | " " | 31 |
|  | Fut9 | Mm_Fut9 | NM_010243.3 | NP_034373.1 | " " | 32 |
|  | Fut10 | Mm_Fut10 | NM_134161.2 | NP_598922.1 | " " | 15, 17 |
|  | Fut11 | Mm_Fut11 | NM_028428.2 | NP_082704.1 | " " | " " |
|  | Fut8 | Mm_Fut8 | NM_016893.4 | NP_058589.2 | α6-FucT (core) | 33 |
|  | Pofut1 | Mm_Pofut1 | NM_080463.3 | NP_536711.3 | O-FucT | 34-36 |
|  | Pofut2 | Mm_Pofut2 | NM_030262.3 | NP_084538.2 | " " | 37 |
| *Danio rerio* (Anamalia, Chordata, Actinopterygii, Cypriniformes, Cyprinidae) | zFT1 | Dr_zFT1 | AB023627.1 | BAA76706.1 | α3-FucT | 38 |
|  | zFT2 | Dr_zFT2 | AB023628.1 | BAA76707.1 | " " | " " |
|  | fut7 | Dr_fut7 | NM_001037390.2 | NP_001032467.1 | " " | 39 |
|  | fut9 | Dr_fut9 | NM_001007454.1 | NP_001007455.1 | " " | " " |
|  | fut10 | Dr_fut10 | AJ879586.1 | CAI52076.1 | " " | 15 |
|  | fut11 | Dr_fut11 | NM_001077174.1 | NP_001070642.1 | " " | " " |
|  | fut8 | Dr_fut8 | NM_001003855.1 | NP_001003855.1 | α6-FucT (core) | 40 |
|  | pofut1 | Dr_pofut1 | NM_205718.2 | NP_991281.2 | O-FucT | - |
|  | pofut2 | Dr_pofut2 | DQ139956.1 | ABA29477.1 | " " | - |
| *Drosophila melanogaster* (Anamalia, Arthropoda, Insecta, Diptera, Drosophilidae) | **FucTA** | Dm_FucTA | NM_140497.2 | NP_648754.2 | α3-FucT (core) | 16, 41, 42 |
|  | **FucTB** | Dm_FucTB | NM_135444.4 | NP_609288.4 | α3-FucT | 16, 42 |
|  | **FucTC** | Dm_FucTC | NM_001042855.2 | NP_001036320.3 | " " | " " |
|  | **FucTD** | Dm_FucTD | NM_138263.2 | NP_612107.1 | " " | 16 |
|  | **FucT6** | Dm_FucT6 | NM_132512.3 | NP_572740.1 | α6-FucT (core) | 16, 41 |
|  | **O-fut1** | Dm_O-fut1 | NM_137087.2 | NP_610931.1 | O-FucT | 16, 43- 45 |
|  | **O-fut2** | Dm_O-fut2 | NM_130560.3 | NP_569916.1 | " " | 16, 46 |
| *Caenorhabditis elegans* (Anamalia, Nematoda, Secernentea, Rhabditida, Rhabditidae) | **CE2FT-1/fut-2** | Ce_2FT-1 | NM_072270.1 | NP_504671.1 | α2-FucT | 47 |
|  | **CE2FT-2** | Ce_2FT-2 | EF015633.1 | ABK20307.1 | " " | 48 |
|  | [CE2FT-F] | Ce_2FT-F | NM_074684.1 | NP_507085.1 | " " | 49 |
|  | [CE2FT-H] | Ce_2FT-H | NM_072271.4 | NP_504672.1 | " " | " " |
|  | [CE2FT-I] | Ce_2FT-I | NM_070852.1 | NP_503253.1 | " " | " " |
|  | [CE2FT-J] | Ce_2FT-J | AF000198.2 | AAB53053.1 | " " | " " |
|  | [CE2FT-K] | Ce_2FT-K | NM_066490.3 | NP_498891.2 | " " | " " |
|  | [CE2FT-L] | Ce_2FT-L | NM_062266.1 | NP_494667.1 | " " | " " |
|  | [CE2FT-M] | Ce_2FT-M | NM_073857.2 | NP_506258.2 | " " | " " |
|  | [CE2FT-N] | Ce_2FT-N | AF024503.2 | AAG24090.4 | " " | " " |
|  | [CE2FT-O] | Ce_2FT-O | NM_070882.1 | NP_503283.1 | " " | " " |
|  | [CE2FT-P] | Ce_2FT-P | NM_070839.2 | NP_503240.2 | " " | " " |
|  | [CE2FT-Q] | Ce_2FT-Q | NM_060694.2 | NP_493095.2 | " " | " " |
|  | [CE2FT-R] | Ce_2FT-R | NM_001026222.1 | NP_001021393.1 | " " | " " |
|  | **fut-1/CEFT-1** | Ce_FT-1 | NM_063461.4 | NP_495862.2 | α3-FucT (core) | 49, 50 |
|  | **fut-5/CEFT-2** | Ce_FT-2 | NM_001027139.1 | NP_001022310.1 | α3-FucT | 51 |
|  | **fut-6/CEFT-3** | Ce_FT-3 | NM_062422.6 | NP_494823.2 | " " | " " |
|  | **fut-3/CEFT-4** | Ce_FT-4 | NM_062705.6 | NP_495106.1 | " " | " " |
|  | **fut-4/CEFT-5** | Ce_FT-5 | NM_061971.3 | NP_494372.2 | " " | " " |
|  | **fut-8** | Ce_fut-8 | NM_072154.4 | NP_504555.2 | α6-FucT (core) | 41 |
|  | **pofut1** | Ce_pofut1 | DQ139948.1 | ABA29469.1 | O-FucT | 52 |
|  | **pad-2** | Ce_pad-2 | NM_171228.1 | NP_741272.1 | " " | 53 |
| *Schistosoma mansoni* (Anamalia, Platyhelminthes, Trematoda, Strigeatida, Schistosomatidae) | **SmFuct** | Sm_FucT-VII | AF016899.1 | AAC27440.1 | α3-FucT | 54 |
|  | **FucTA** | Sm_FucTA | AF183577.1 | AAF71198.1 | " " | 55 |
|  | FucTB | Sm_FucTB | GU574750.1 | ADO17522.1 | " " | Present study |
|  | FucTC | Sm_FucTC | GU574751.1 | ADO17523.1 | " " | " " |
|  | FucTD | Sm_FucTD | GU574752.1 | ADO17524.1 | " " | " " |
|  | FucTE | Sm_FucTE | GU574753.1 | ADO17525.1 | " " | " " |
|  | FucTF | Sm_FucTF | GU574754.1 | ADO17526.1 | " " | " " |
|  | FucTG | Sm_FucTG | GU574755.1 | - | α3-FucT (pseudo) | " " |
|  | FucTH | Sm_FucTH | JN406372.1 | AEW22968.1 | α6-FucT (core) | " " |
|  | FucTI | Sm_FucTI | JN406373.1 | AEW22969.1 | " " | " " |
|  | FucTJ | Sm_FucTJ | JN406374.1 | AEW22970.1 | " " | " " |
|  | FucTK | Sm_FucTK | JN406375.1 | AEW22971.1 | " " | " " |
|  | FucTL | Sm_FucTL | JN406376.1 | AEW22972.1 | " " | " " |
|  | FucTM | Sm_FucTM | JN406377.1 | AEW22973.1 | " " | " " |
|  | POFucTA | Sm_POFucTA | JN406378.1 | AEW22974.1 | O-FucT | " " |
|  | POFucTB | Sm_POFucTB | JN406379.1 | AEW22975.1 | " " | " " |
| *Schistosoma japonicum* (Anamalia, Platyhelminthes, Trematoda, Strigeatida, Schistosomatidae) | FUT8 | CAX72936.1 | FN317205.1 | CAX72936.1 | α6-FucT | - |
|  | - | CAX73054.1 | FN317323.1 | CAX73054.1 | α6-FucT | - |
|  | - | Sjp_0036210 | Sjp_0036210 ^e^ | Sjp_0036210 ^e^ | α3-FucT | - |
| *Dictyostelium discoideum* (Protozoa*, Mycetozoa, Dictyosteliomycetes, Dictyosteliales, Dictyosteliaceae) | **pgtA/fucB** | Dd_pgtA | AF279134.1 | AAF82378.1 | β3-GalT/α2-FucT (dual) | 56, 57 |

^a^ Taxa were obtained from the Catalogue of Life: 2011 Annual Checklist [58]. Asterisks (*) indicate unofficial nomenclature.

^b^ Official gene names/identifiers (when available) are provided; bracketed names refer to nomenclature used in the cited reference. Gene names in boldface type were used as query sequences to identify homologs in the ShistoDB [59].

^c^ α2-FucT, α2-fucosyltransferase; α3-FucT, α3-fucosyltransferase; α3/4-FucT, bifunctional α3/α4-fucosyltransferase; α6-FucT, α6-fucosyltransferase; O-FucT, protein O-fucosyltransferase; β3-GalT/α2-FucT, bifunctional β3-galactosyltransferase/α2-fucosyltransferase

^d^ "core", N-glycan chitobiose core fucosylation activity; “pseudo”, pseudogene; “dual”, dual-function glycosyltransferase

^e^ Sequence was obtain from SchistoDB and is referenced by annotation number.

# References

1. Larsen RD, Ernst LK, Nair RP, Lowe JB (1990) Molecular cloning, sequence, and expression of a human GDP-L-fucose:beta-D-galactoside 2-alpha-L-fucosyltransferase cDNA that can form the H blood group antigen. Proc Natl Acad Sci USA 87: 6674-6678.
2. Koda Y, Soejima M, Kimura H (1997) Structure and expression of H-type GDP-L-fucose:β-D-galactoside 2-α-L-fucosyltransferase gene (FUT1). Two transcription start sites and alternative splicing generate several forms of FUT1 mRNA. J Biol Chem 272: 7501–7505.
3. Kelly RJ, Rouquier S, Giorgi D, Lennon GG, Lowe JB (1995) Sequence and expression of a candidate for the human Secretor blood group α(1,2)fucosyltransferase gene (FUT2). Homozygosity for an enzyme-inactivating nonsense mutation commonly correlates with the non-secretor phenotype. J Biol Chem 270: 4640-4649.
4. Koda Y, Soejima M, Wang B, Kimura H (1997) Structure and expression of the gene encoding secretor-type galactoside 2-α-L-fucosyltransferase (FUT2). Eur J Biochem 246: 750-755.
5. Dupuy F, Petit JM, Mollicone R, Oriol R, Julien R, Maftah A (1999) A single amino acid in the hypervariable stem domain of vertebrate α1,3/1,4-fucosyltransferases determines the type 1/type 2 transfer. Characterization of acceptor substrate specificity of the lewis enzyme by site-directed mutagenesis. J Biol Chem 274: 12257–12262.
6. Dupuy F, Germot A, Julien R, Maftah A (2004) Structure/function study of Lewis α3- and α3/4-fucosyltransferases: the α1,4 fucosylation requires an aromatic residue in the acceptor-binding domain. Glycobiology 14: 347-356.
7. Kukowska-Latallo JF, Larsen RD, Nair RP, Lowe JB (1990) A cloned human cDNA determines expression of a mouse stage-specific embryonic antigen and the Lewis blood group α(1,3/1,4)fucosyltransferase. Genes Dev 4: 1288-1303.
8. Holmes EH, Yen TY, Thomas S, Joshi R, Nguyen A, Long T, Gallet F, Maftah A, Julien R, Macher BA (2000) Human α1,3/4 fucosyltransferases. Characterization of highly conserved cysteine residues and N-linked glycosylation sites. J Biol Chem 275: 24237–24245.
9. Lowe JB, Kukowska-Latallo JF, Nair RP, Larsen RD, Marks RM, Macher BA, Kelly RJ, Ernst LK (1991) Molecular cloning of a human fucosyltransferase gene that determines expression of the Lewis x and VIM-2 epitopes but not ELAM-1-dependent cell adhesion. J Biol Chem 266: 17467-17477.
10. Weston BW, Nair RP, Larsen RD, Lowe JB (1992) Isolation of a novel human α(1,3)fucosyltransferase gene and molecular comparison to the human Lewis blood group α(1,3/1,4)fucosyltransferase gene. Syntenic, homologous, nonallelic genes encoding enzymes with distinct acceptor substrate specificities. J Biol Chem 267: 4152-4160.
11. Koszdin KL, Bowen BR (1992) The cloning and expression of a human α-1,3 fucosyltransferase capable of forming the E-selectin ligand. Biochem Biophys Res Commun 187: 152-157.
12. Weston BW, Smith PL, Kelly RJ, Lowe J (1992) Molecular cloning of a fourth member of a human α(1,3)fucosyltransferase gene family. Multiple homologous sequences that determine expression of the Lewis x, sialyl Lewis x, and difucosyl sialyl Lewis x epitopes. J Biol Chem 267: 24575-24584.
13. Natsuka S, Gersten KM, Zenita K, Kannagi R, Lowe JB (1994) Molecular cloning of a cDNA encoding a novel human leukocyte α-1,3-fucosyltransferase capable of synthesizing the sialyl Lewis x determinant. J Biol Chem 269: 16789-16794.
14. Kaneko M, Kudo T, Iwasaki H, Ikehara Y, Nishihara S, Nakagawa S, Sasaki K, Shiina T, Inoko H, Saitou N, Narimatsu H (1999) α1,3-Fucosyltransferase IX (Fuc-TIX) is very highly conserved between human and mouse; molecular cloning, characterization and tissue distribution of human Fuc-TIX. *FEBS Lett* 452: 237-242.
15. Mollicone R, Moore SE, Bovin N, Garcia-Rosasco M, Candelier JJ, Martinez-Duncker I, Oriol R (2009) Activity, splice variants, conserved peptide motifs, and phylogeny of two new α1,3-fucosyltransferase families (FUT10 and FUT11). J Biol Chem 284: 4723–4738.
16. Roos C, Kolmer M, Mattila P, Renkonen R (2002) Composition of *Drosophila melanogaster* proteome involved in fucosylated glycan metabolism. J Biol Chem 277: 3168-3175.
17. Baboval T, Smith FI (2002) Comparison of human and mouse Fuc-TX and Fuc-TXI genes, and expression studies in the mouse. Mamm Genome 13: 538-541.
18. Takahashi T, Ikeda Y, Tateishi A, Yamaguchi Y, Ishikawa M, Taniguchi N (2000) A sequence motif involved in the donor substrate binding by α1,6-fucosyltransferase: the role of the conserved arginine residues. Glycobiology 10: 503-510.
19. Yanagidani S, Uozumi N, Ihara Y, Miyoshi E, Yamaguchi N, Taniguchi N (1997) Purification and cDNA cloning of GDP-L-Fuc:N-acetyl-beta-D-glucosaminide:α1-6 fucosyltransferase (α1-6 FucT) from human gastric cancer MKN45 cells. J Biochem 121: 626-632.
20. Martinez-Duncker I, Michalski JC, Bauvy C, Candelier JJ, Mennesson B, Codogno P, Oriol R, Mollicone R (2004) Activity and tissue distribution of splice variants of α6-fucosyltransferase in human embryogenesis. Glycobiology 14: 13-25.
21. Wang Y, Shao L, Shi S, Harris RJ, Spellman MW, Stanley P, Haltiwanger RS (2001) Modification of epidermal growth factor-like repeats with O-fucose. Molecular cloning and expression of a novel GDP-fucose protein O-fucosyltransferase. J Biol Chem 276: 40338–40345.
22. Shi S, Stanley P (2003) Protein O-fucosyltransferase 1 is an essential component of Notch signaling pathways. Proc Natl Acad Sci USA 100: 5234–5239.
23. Martinez-Duncker I, Mollicone R, Candelier JJ, Breton C, Oriol R (2003) A new superfamily of protein-O-fucosyltransferases, α2-fucosyltransferases, and α6-fucosyltransferases: phylogeny and identification of conserved peptide motifs. Glycobiolog*y* 13: 1C-5C.
24. Domino SE, Hiraiwa N, Lowe JB (1997) Molecular cloning, chromosomal assignment and tissue-specific expression of a murine α(1,2)fucosyltransferase expressed in thymic and epididymal epithelial cells. Biochem J 327: 105-115.
25. Domino SE, Zhang L, Lowe JB (2001) Molecular cloning, genomic mapping, and expression of two secretor blood group α(1,2)fucosyltransferase genes differentially regulated in mouse uterine epithelium and gastrointestinal tract. J Biol Chem 276: 23748–23756.
26. Lin B, Saito M, Sakakibara Y, Hayashi Y, Yanagisawa M, Iwamori M (2001) Characterization of three members of murine α1,2-fucosyltransferases: change in the expression of the Se gene in the intestine of mice after administration of microbes. Arch Biochem Biophys 388: 207–215.
27. Lin B, Hayashi Y, Saito M, Sakakibara Y, Yanagisawa M, Iwamori M (2000) GDP-fucose: β-galactoside α1,2-fucosyltransferase, MFUT-II, and not MFUT-I or -III, is induced in a restricted region of the digestive tract of germ-free mice by host-microbe interactions and cycloheximide. Biochim Biophys Acta 1487: 275-285.
28. Lin B, Makiko S, Masao I (2005) Substrate specificity of three murine GDP-fucose: β-galactoside α1,2-fucosyltransferases. Zhongguo Yi Xue Ke Xue Yuan Xue Bao 27: 761-766.
29. Gersten KM, Natsuka S, Trinchera M, Petryniak B, Kelly RJ, Hiraiwa N, Jenkins NA, Gilbert DJ, Copeland NG, Lowe JB (1995) Molecular cloning, expression, chromosomal assignment, and tissue-specific expression of a murine α-(1,3)-fucosyltransferase locus corresponding to the human ELAM-1 ligand fucosyl transferase. J Biol Chem 270: 25047–25056.
30. Ozawa M, Muramatsu T (1996) Molecular cloning and expression of a mouse α-1,3 fucosyltransferase gene that shows homology with the human α-1,3 fucosyltransferase IV gene. J Biochem 119: 302-308.
31. Smith PL, Gersten KM, Petryniak B, Kelly RJ, Rogers C, Natsuka Y, Alford JA 3rd, Scheidegger EP, Natsuka S, Lowe JB (1996) Expression of the α(1,3)fucosyltransferase Fuc-TVII in lymphoid aggregate high endothelial venules correlates with expression of L-selectin ligands. J Biol Chem 271: 8250–8259.
32. Kudo T, Ikehara Y, Togayachi A, Kaneko M, Hiraga T, Sasaki K, Narimatsu H (1998) Expression cloning and characterization of a novel murine α1,3-fucosyltransferase, mFuc-TIX, that synthesizes the Lewis x (CD15) epitope in brain and kidney. J Biol Chem 273: 26729–26738.
33. Hayashi H, Yoneda A, Asada M, Ikekita M, Imamura T (2000) Molecular cloning of mouse α-1,6-fucosyltransferase and expression of its mRNA in the developing cerebrum. DNA Seq 11: 91-96.
34. Luo Y, Haltiwanger RS (2005) O-fucosylation of notch occurs in the endoplasmic reticulum. J Biol Chem 280: 11289–11294.
35. Stahl M, Uemura K, Ge C, Shi S, Tashima Y, Stanley P (2008) Roles of Pofut1 and O-fucose in mammalian Notch signaling. J Biol Chem 283: 13638–13651.
36. Yao D, Huang Y, Huang X, Wang W, Yan Q, Wei L, Xin W, Gerson S, Stanley P, Lowe JB, Zhou L (2011) Protein O-fucosyltransferase 1 (Pofut1) regulates lymphoid and myeloid homeostasis through modulation of Notch receptor ligand interactions. Blood 117: 5652-5662.
37. Du J, Takeuchi H, Leonhard-Melief C, Shroyer KR, Dlugosz M, Haltiwanger RS, Holdener BC (2010) O-fucosylation of thrombospondin type 1 repeats restricts epithelial to mesenchymal transition (EMT) and maintains epiblast pluripotency during mouse gastrulation. Dev Biol 346: 25-38.
38. Kageyama N, Natsuka S, Hase S (1999) Molecular cloning and characterization of two zebrafish α(1,3)fucosyltransferase genes developmentally regulated in embryogenesis. J Biochem 125: 838-845.
39. Petit D, Maftah A, Julien R, Petit JM (2006) En bloc duplications, mutation rates, and densities of amino acid changes clarify the evolution of vertebrate α-1,3/4-fucosyltransferases. J Mol Evol 63: 353–364.
40. Seth A, Machingo QJ, Fritz A, Shur BD (2010 ) Core fucosylation is required for midline patterning during zebrafish development. Dev Dyn 239: 3380–3390.
41. Paschinger K, Staudacher E, Stemmer U, Fabini G, Wilson IB (2005) Fucosyltransferase substrate specificity and the order of fucosylation in invertebrates. Glycobiology 15: 463-474.
42. Fabini G, Freilinger A, Altmann F, Wilson IB (2001) Identification of core α1,3-fucosylated glycans and cloning of the requisite fucosyltransferase cDNA from *Drosophila melanogaster*. Potential basis of the neural anti-horseadish peroxidase epitope. J Biol Chem 276: 28058–28067.
43. Okajima T, Xu A, Irvine KD (2003) Modulation of notch-ligand binding by protein O-fucosyltransferase 1 and fringe. J Biol Chem 278: 42340-42345.
44. Sasamura T, Ishikawa HO, Sasaki N, Higashi S, Kanai M, Nakao S, Ayukawa T, Aigaki T, Noda K, Miyoshi E, Taniguchi N, Matsuno K (2007) The O-fucosyltransferase O-fut1 is an extracellular component that is essential for the constitutive endocytic trafficking of Notch in *Drosophila*. Development 134: 1347-1356.
45. Okajima T, Irvine KD (2002) Regulation of notch signaling by o-linked fucose. Cell 111: 893-904.
46. Luo Y, Koles K, Vorndam W, Haltiwanger RS, Panin VM (2006) Protein O-fucosyltransferase 2 adds O-fucose to thrombospondin type 1 repeats. J Biol Chem 281: 9393–9399.
47. Zheng Q, van Die I, Cummings RD (2002) Molecular cloning and characterization of a novel α1,2-fucosyltransferase (CE2FT-1) from *Caenorhabditis elegans*. J Biol Chem 277: 39823–39832.
48. Zheng Q, van Die I, Cummings RD (2008) A novel α1,2-fucosyltransferase (CE2FT-2) in *Caenorhabditis elegans* generates H-type 3 glycan structures. Glycobiology 18: 290–302.
49. Oriol R, Mollicone R, Cailleau A, Balanzino L, Breton C (1999) Divergent evolution of fucosyltransferase genes from vertebrates, invertebrates, and bacteria. Glycobiology 9: 323-334.
50. Paschinger K, Rendic D, Lochnit G, Jantsch V, Wilson IB (2004) Molecular basis of anti-horseradish peroxidase staining in *Caenorhabditis elegans*. J Biol Chem 279: 49588–49598.
51. Nguyen K, van Die I, Grundahl KM, Kawar ZS, Cummings RD (2007) Molecular cloning and characterization of the *Caenorhabditis elegans* α1,3-fucosyltransferase family. Glycobiology 17: 586-599.
52. Loriol C, Dupuy F, Rampal R, Dlugosz MA, Haltiwanger RS, Maftah A, Germot A (2006) Molecular evolution of protein O-fucosyltransferase genes and splice variants. Glycobiology 16: 736–747.
53. Menzel O, Vellai T, Takacs-Vellai K, Reymond A, Mueller F, Antonarakis SE, Guipponi M (2004) The *Caenorhabditis elegans* ortholog of C21orf80, a potential new protein O-fucosyltransferase, is required for normal development. Genomics 84: 320–330.
54. Marques ET, Weiss JB, Strand M (1998) Molecular characterization of a fucosyltransferase encoded by *Schistosoma mansoni*. Mol Biochem Parasitol 93: 237-250.
55. Trottein F, Mollicone R, Fontaine J, de Mendonca R, Pillar F, Pierce R, Oriol R, Capron M (2000) Molecular cloning of a putative alpha3-fucosyltransferase from *Schistosoma mansoni*. Mol Biochem Parasitol 107: 279-287.
56. van der Wel H, Morris HR, Panico M, Paxton T, North SJ, Dell A, Thomson JM, West CM (2001) A non-Golgi α1,2-fucosyltransferase that modifies Skp1 in the cytoplasm of *Dictyostelium*. J Biol Chem 276: 33952–33963.
57. van der Wel H, Fisher SZ, West CM: A bifunctional diglycosyltransferase forms the Fucα1,2Galβ1,3-disaccharide on Skp1 in the cytoplasm of *Dictyostelium*. *J Biol Chem* 2002, 277:46527–46534.
58. Catalogue of Life: 2011 Annual Checklist [http://www.catalogueoflife.org]
59. Zerlotini A, Heiges M, Wang H, Moraes RL, Dominitini AJ, Ruiz JC, Kissinger JC, Oliveira G (2009) SchistoDB: a *Schistosoma mansoni* genome resource. Nucleic Acids Res 37: D579-582.
